# Soft subdermal implant capable of wireless battery charging and programmable controls for applications in optogenetics

**DOI:** 10.1038/s41467-020-20803-y

**Published:** 2021-01-22

**Authors:** Choong Yeon Kim, Min Jeong Ku, Raza Qazi, Hong Jae Nam, Jong Woo Park, Kum Seok Nam, Shane Oh, Inho Kang, Jae-Hyung Jang, Wha Young Kim, Jeong-Hoon Kim, Jae-Woong Jeong

**Affiliations:** 1 School of Electrical Engineering, Korea Advanced Institute of Science and Technology, Daejeon, Republic of Korea; 2 Department of Physiology, Graduate School of Medical Science, Yonsei University College of Medicine, Seoul, Republic of Korea; 3 Department of Electrical, Computer, and Energy Engineering, University of Colorado, Boulder, CO USA; 4 Department of Chemical and Biomolecular Engineering, Yonsei University, Seoul, Republic of Korea; 5 Department of Physiology, Yonsei University College of Medicine, Seoul, Republic of Korea

**Keywords:** Optogenetics, Biomedical engineering, Electrical and electronic engineering

## Abstract

Optogenetics is a powerful technique that allows target-specific spatiotemporal manipulation of neuronal activity for dissection of neural circuits and therapeutic interventions. Recent advances in wireless optogenetics technologies have enabled investigation of brain circuits in more natural conditions by releasing animals from tethered optical fibers. However, current wireless implants, which are largely based on battery-powered or battery-free designs, still limit the full potential of in vivo optogenetics in freely moving animals by requiring intermittent battery replacement or a special, bulky wireless power transfer system for continuous device operation, respectively. To address these limitations, here we present a wirelessly rechargeable, fully implantable, soft optoelectronic system that can be remotely and selectively controlled using a smartphone. Combining advantageous features of both battery-powered and battery-free designs, this device system enables seamless full implantation into animals, reliable ubiquitous operation, and intervention-free wireless charging, all of which are desired for chronic in vivo optogenetics. Successful demonstration of the unique capabilities of this device in freely behaving rats forecasts its broad and practical utilities in various neuroscience research and clinical applications.

## Abstract (web summary)

Although wireless optogenetic technologies enable brain circuit investigation in freely moving animals, existing devices have limited their full potential, requiring special power setups. Here, the authors report fully implantable optogenetic systems that allow intervention-free wireless charging and controls for operation in any environment.

## Introduction

Unveiling the working mechanisms of the brain can open new opportunities for the treatment of brain disorders and neurodegenerative diseases^1–5^. Optogenetics^6^—using light to engage biological systems with exogenously expressed light-sensitive proteins—is an emerging neuroscience tool, which can modulate neuronal populations in a highly selective way. This powerful technique allows precise activation or inhibition of specific types of neurons, thus providing the ability to explore neuronal functions and related signal pathways at the circuit level in the central and peripheral nervous systems^7^. However, conventional approaches for optogenetics involve tethered optical fibers for light delivery, which significantly restrict animals’ movement, cause increased inflammation in soft brain tissue owing to their rigid mechanics, and lack scalable control capability for in vivo studies involving multiple animals^8,9^. Advancements in materials and micro/nanofabrication techniques have enabled ultrathin neurophilic probes^10–12^ and multifunctional polymeric fibers^13,14^ that allow chronic biocompatible integration with neural tissue, but they still rely on leashed setups with bulky equipment, thus restricting their full capabilities.

Recent developments of wireless optogenetic devices have tried to abate limitations associated with the tethered approach^9,15–27^. Current wireless technologies largely rely on battery-powered^15–19^ or battery-free approaches^20–27^. Battery-powered devices provide a stable stand-alone power solution but require intermittent replacement of batteries for continuous operation, thereby necessitating head-mounted configurations vulnerable to external stress^15–19^. Battery-free implants with miniaturized radiofrequency (RF) energy-harvesting circuits, on the other hand, overcome this limitation by allowing their full implantation inside the body^22–27^. However, their wireless operation is susceptible to angular orientations, does not support selective control among multiple animals mingled together, and most importantly, always requires special bulky cages equipped with an RF power transfer system. These features substantially constrain diverse behavioral experiment setups for complex neuroscience research and frustrate possible future use of this technology in daily human life for therapeutic interventions. Some recent advances have tried to combine batteries with a wireless energy-harvesting module in implantable systems to enable wireless charging of batteries. However, their bulky and rigid configurations limit biomechanically compatible chronic use within the body, and moreover, the wireless charging capability in freely moving animals has not been demonstrated^28,29^.

To overcome these challenges and maximize the use of wireless optogenetics, we present a fully implantable, soft, wirelessly rechargeable optoelectronic systems that can be conformally integrated within the body and can be easily controlled by a readily available smartphone. This implant combines the advantages of both state-of-the-art battery-powered (head-mounted) and battery-free (fully implantable) devices while overcoming the fundamental limitations of each approach (see Supplementary Table 1 for detailed comparison with contemporary technologies). More specifically, our integrated devices are powered by a subcutaneously implanted, wirelessly rechargeable battery and controlled using bluetooth low energy (BLE) wireless technology. This combination allows not only minimalistic experimental setups without requiring special cages for reliable device operation, but also enables highly versatile wireless controls such as simultaneous selectivity within multiple animal cohorts, omnidirectional wireless operation, and closed-loop control. Furthermore, the design incorporating a wirelessly rechargeable battery system completely eliminates the need for disruptive periodic surgeries for replacement of batteries, which is a big pain stake for patients, implanted with battery-operated devices such as deep brain stimulators and pacemakers, thus allowing intervention-less chronic in vivo operation. Our in vivo studies with freely behaving animals and phantom human models demonstrate the broad utility and immense potential of wirelessly rechargeable implants for neuroscience research and clinical applications.

## Results

### Design and operational principles

**Fig. 1:**
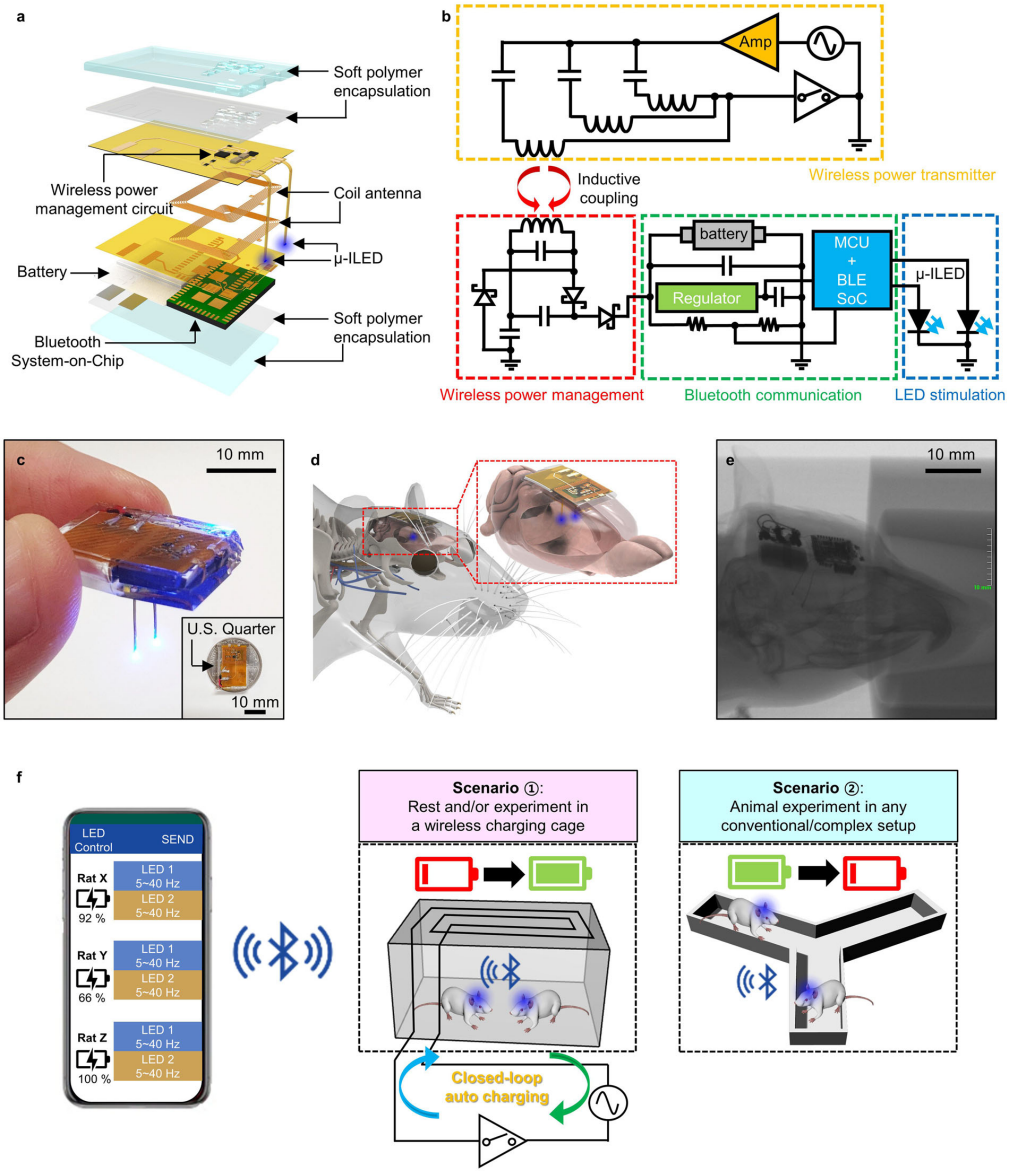
Design and working principles of fully implantable, wireless rechargeable, soft optoelectronic systems for in vivo optogenetics. **a** Exploded view schematic diagram of a soft wireless optoelectronic system with bilateral probes, consisting of microscale inorganic light-emitting diodes (μ-ILEDs), a power management circuit, radiofrequency (RF) coil antennas, a battery, and a Bluetooth Low Energy System-on-Chip (BLE SoC). **b** Electrical circuit diagram of the overall power regulation system, which consists of a wireless power transmitter and a wireless rechargeable optoelectronic system (i.e., wireless receiver). **c** Optical image of a wireless optoelectronic system held with fingers. The inset shows that the device is smaller than a US quarter. **d** Conceptual illustration of a wireless optoelectronic probe system subcutaneously implanted in a rodent head for control of neural circuits deep in the brain. The inset highlights conformal integration of the device with a rat brain. **e** X-ray image of a rat implanted with a wireless optoelectronic system. **f** Operation principles of wireless optoelectronic systems in two different scenarios. The wireless implants can operate in (i) a cage equipped with a closed-loop RF auto-charging system for chronic in vivo study, or (ii) any conventional experimental setup without needing a special RF power transmitter, using the integrated battery. In all cases, a custom-designed smartphone app allows user-friendly control of the wireless implants.

Figure 1a shows an exploded view schematic diagram of a wirelessly rechargeable, smartphone-controlled optoelectronic system (see the Methods section and Supplementary Fig. 1 for fabrication details). The wireless optoelectronic system consists of four main functional parts: (i) optoelectronic neural probes for photostimulation, (ii) a power management circuit with a flexible coil antenna and a rechargeable Lithium Polymer (LiPo) battery (GMB-300910, PowerStream Technology) for wireless charging and operation, (iii) Bluetooth Low Energy System-on-Chip (BLE SoC; RFD77101, RF Digital Corporation) for wireless control, and (iv) soft polymer encapsulation for biocompatible device packaging. The power management circuit with a coil antenna primarily helps harvest wireless RF energy to form DC electrical charging currents for the LiPo battery (12 mAh, 0.3 g). The coil antenna (16 turns distributed on two interconnected layers) is constructed by patterning copper traces (35 μm thick) on thin polyimide (PI; 25 μm thick) layers. The bilateral neural probes integrate microscale inorganic light-emitting diodes (μ-ILEDs; blue (470 nm), 270 × 220 × 50 μm^3^; Supplementary Fig. 2), which are controlled by the BLE SoC to enable wireless optogenetic modulation in both left and right sides of the brain. Each probe is 100 μm-thick and 300 μm-wide, making its cross-sectional area similar to that of single-mode optical fibers (0.03 mm^2^ for an optoelectronic probe vs. 0.042 mm^2^ for an optical fiber). Previous studies demonstrated biocompatibility and long-term in vivo stability of this type of optoelectronic probes in brain tissue^17,19,20,23,24^. Soft polymer encapsulation has a key role in not only providing device protection from bio-fluids and external shock, but also allowing conformal bio-integration for adaptive and reliable operation inside the body. The encapsulation consists of multiple polymer layers. The inner encapsulation layers are composed of polydimethylsiloxane (PDMS; 600 μm thick) and Parylene C (7 μm thick, 0.083 g mm m^–2^ day^–1^ water vapor permeability) and work as a protection barrier against biofluid, while the outer ultrasoft polymer (33.4 kPa, 1400 μm thick; Ecoflex GEL, Smooth-On Inc.) offers a biocompatible mechanical buffer for seamless chronic integration with tissue (Supplementary Fig. 3). All the materials and electronic components are commercially available and can be processed and assembled using standard fabrication techniques, thus facilitating mass production and deployment of devices for neuroscience research.

The circuit diagram of the overall wireless system is illustrated in Fig. 1b. Both a wireless power transmitter (Fig. 1b, top) and a wireless receiver (i.e., wireless optoelectronic device; Fig. 1b, bottom) are designed to match the resonant frequency at 6.78 MHz, following the Alliance for Wireless Power (A4WP) standard, which is broadly used for simultaneous wireless charging of multiple devices. As the coil antenna of the device receives the wirelessly transmitted power via inductive coupling, it supplies a rectified and multiplied voltage to the battery for energy harvesting through a voltage-doubler circuit. To prevent undesired discharging of the battery by reverse-flow of the current, the battery located at the load is connected with a Schottky diode in series. The wirelessly charged battery then delivers stable DC power to BLE SoC and μ-ILEDs for their reliable wireless operation.

Figure 1c–f highlights various features and operational concepts of fully implantable, wirelessly rechargeable optoelectronic systems. The compact and lightweight electronic design (Fig. 1c; 1.4 g; 19 mm-long × 12 mm-wide × 5 mm thick) allows seamless integration of the device inside the body of small animals such as rodents and allows their undisturbed naturalistic behavior and movement (Fig. 1d and e and Supplementary Fig. 4). The former feature of the device, when compared with contemporary head-mounted systems^15–21^, substantially reduces the risk of device damage and unwanted stress on the tissue where the device is implanted, which may be caused by heavy interactions between animals especially in group housing conditions and/or by chance of bumping the device on rigid cages during free movement. In addition, the approach of integrating wireless power transfer with a rechargeable battery provides unique attributes that make it surpass existing battery-powered and battery-free wireless technologies. One of the most important features of this design is that wireless recharging capability completely removes the need for intermittent replacement of batteries, opening opportunities for non-disruptive chronic in-body operation. Furthermore, powered by an integrated battery, the device enables operations independent of environment and power setup, making their use more versatile. Some possible scenarios of its use in behavioral neuroscience research are illustrated in Fig. 1f. The devices can be wirelessly charged while animals move freely in a home cage equipped with a wireless closed-loop auto-charging system. Once devices are fully charged, animals can be placed in “any” experimental setup (i.e., no need for a power transfer setup), thereby facilitating their wide deployment for numerous neuroscience studies. In all cases, using a custom-designed smartphone app, μ-ILED operation (5−40 Hz with 10 ms pulse width) can be controlled wirelessly, and the battery level can be monitored in real-time through BLE communication (Fig. 1f, Supplementary Fig. 5, and Supplementary Movie 1). BLE is an attractive wireless control scheme for neuroscience research, which overcomes limitations of both IR^15,17,18^ and other RF wireless controls^20–27^. Some of the advantages of BLE control include highly selective control of single or multiple animals within the vicinity, no line-of-sight handicap, long working distance (up to ~100 m), and bidirectional communication that enables closed-loop control, as demonstrated in our simulation experiment (Supplementary Fig. 6). All the aforementioned characteristics make this tool highly versatile and potent option for chronic in vivo applications in neuroscience research.

### Electrical characteristics of wireless battery charging systems

**Fig. 2:**
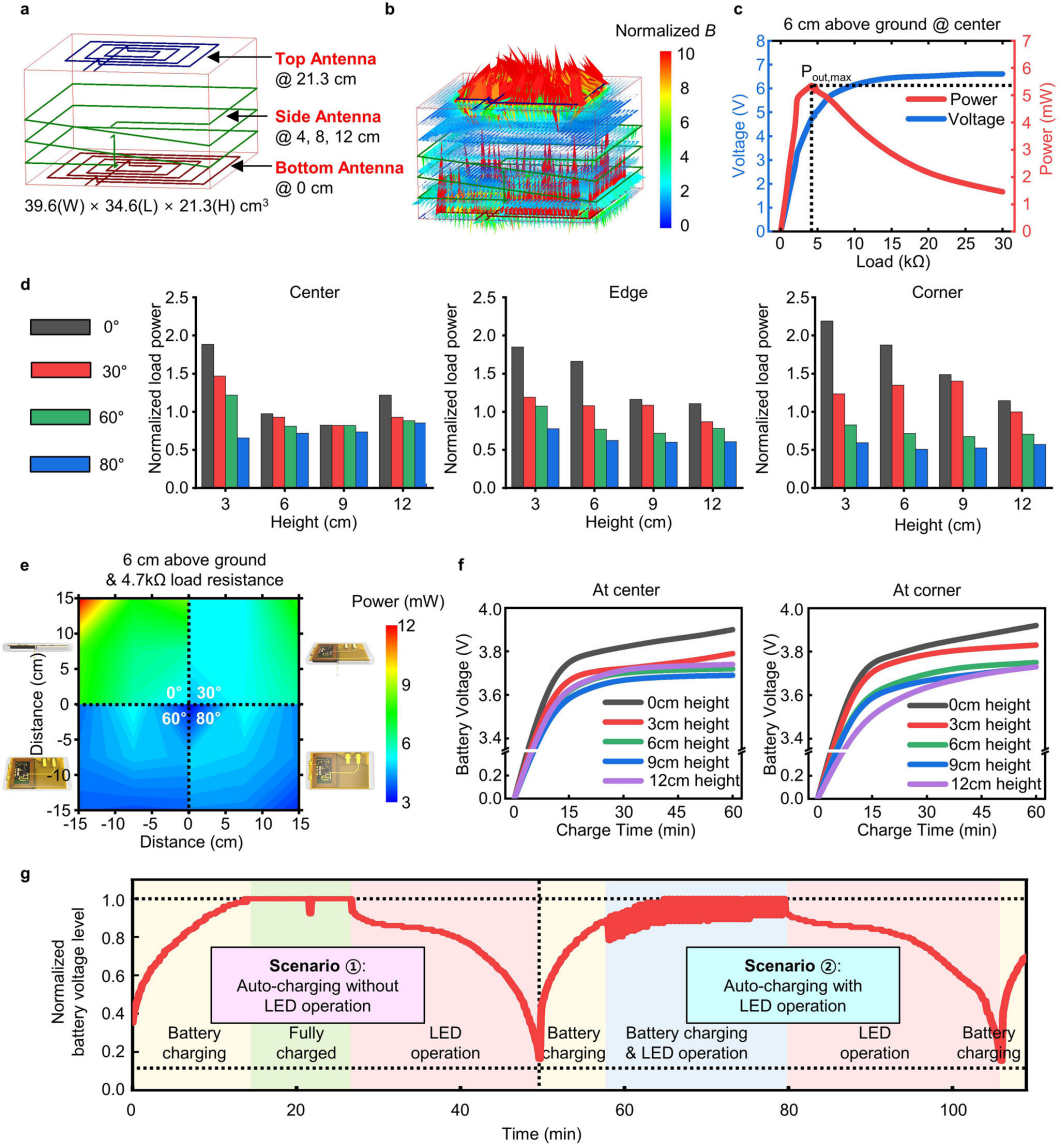
Electrical characteristics of wireless rechargeable optoelectronic systems. **a**, **b** Schematic diagram (**a**) and simulated magnetic field density (*B*) (**b**) of a rat cage (39.6 cm (*W*) × 34.6 cm (*L*) × 21.3 cm (*H*)) with three loop antennas (top, side, and bottom antenna). **c** Measurement of rectified voltage (blue line) and power delivered to the load (red line) of a wireless device with different load resistances (from 7–30 kΩ), which was placed at the center with a 6 cm height above the ground of the rat cage. The maximum output power (~5.3 mW) was obtained with a 4.7 kΩ load resistance, when an input power of 12.5 W was transmitted. **d** Normalized power at the output load (4.7 kΩ) of wireless devices at various locations (center, edge, and corner), heights (3, 6, 9, and 12 cm) and orientations (0°, 30°, 60°, and 80°) inside a rat cage. **e** Variation of energy-harvesting efficiency with respect to location and angular orientation of a device at a height of 6 cm in the rat cage. **f** Wireless battery charging characteristics in saline water (0.9%) at various heights (0, 3, 6, 9, and 12 cm) for the device located at the center (left) and the corner (right) of the cage. Battery charging was started after the battery was fully discharged. **g** Real-time monitoring of the battery level during the wireless closed-loop auto-charging operation of the optoelectronic system in two different sequential scenarios: wireless auto-charging (i) without and (іі) with LED operation. When the battery voltage level reaches the preset maximum, charging is automatically turned off temporarily for 10 s to confirm whether the battery is actually fully charged (see small dimple in the recorded signal at 22 min). The small fluctuation of the battery voltage level appearing between 58 and 80 min is due to electrical noise caused by the LED operation. The proof-of-principle operation was carried out with a device immersed in saline water (0.9%), which was located at the center of the rat cage floor.

The soft optoelectronic implants can be recharged wirelessly via inductive coupling at 6.78 MHz, while animals stay in their native home cages. Figure 2 shows the electrical characteristics of the wireless battery charging systems under various operational conditions in a typical rat cage (39.6 × 34.6 × 21.3 cm^3^) installed with three RF coil antennas located at the top (blue, 21.3 cm height), side (green, heights of 4, 8, and 12 cm), and bottom (red, 0 cm height) (Fig. 2a). The antennas are designed to be integrated with a rat cage through a simple assembly process (Supplementary Fig. 7) to allow facile and rapid setup for different cages with the same dimensions. In the RF transmitter design, the side antenna alone or the top and bottom antennas without the side antenna are not sufficient for effective wireless charging of the devices implanted in freely moving rodents due to either relatively weak magnetic field generation (Supplementary Fig. 8a) or field vacancy in 3D space (Supplementary Fig. 8b), respectively. By operating all three antennas simultaneously, the wireless charging system can supply magnetic fields strong enough to cover all over the 3D space within the cage (Fig. 2b, Supplementary Fig. 8c, and Supplementary Movie 2), thereby supporting omni-locational energy harvesting.

Wireless optoelectronic devices (i.e., receivers) are designed to effectively scavenge transmitted RF power regardless of their locations and angles within the cage. Figure 2c shows the rectified voltage and the delivered power to the load of a wireless device with different load resistances at the cage center (6 cm height) when 12.5 W input power is supplied to the transmit loop antennas of the cage. The peak delivered power (~5.3 mW) can be obtained at a voltage of ~5 V, which is achieved by rectification and multiplication through the wireless power management circuit (Fig. 1b, red dotted box). Overall, these devices show some degree of variation in wirelessly received power depending on their locations (center, edge, and corner), heights (3 cm, 6 cm, 9 cm, and 12 cm above the ground), and angular orientations (0°, 30°, 60°, and 80° with respect to horizontal plane) inside the cage, demonstrating a clear inverse relationship between the angular orientation and the received power across the 3D space (Fig. 2d, e). However, there is no dramatic decrease in energy-harvesting efficiency even at very high angular orientation (80°) or with height changes towards the middle space of the cage, thereby allowing stable wireless charging of the battery. This is owing to the combinatorial antenna design setup that combines fields from the top, the side, and the bottom coils to offer effective field coverage in space and direction within the cage.

A proof-of-concept experiment verifies the device capabilities for wireless charging. To simulate in vivo operation, we immersed the device in saline water (0.9%) and characterized the battery charging behavior at various locations inside the cage (Fig. 2f and Supplementary Fig. 7b). For all cases, 45 min was enough to charge the battery (~3.7 V) to operate a μ-ILED at 5 − 40 Hz with a 10 ms pulse width for >40 min (Supplementary Fig. 9a, b). This means that charged devices can be operated anywhere and anytime without relying on the power transfer setup anymore, thus overcoming the critical limitation of current implantable, battery-free wireless device technologies^22–27^. Note that the device operation time can be further increased by employing a battery with a larger capacity and/or a more advanced low power BLE SoC (Supplementary Fig. 9c, e).

The optoelectronic devices can also be automatically charged wirelessly through a closed-loop system integrated with the RF power transmitter (Fig. 1b, yellow dotted box). The wireless auto-charging system (Fig. 1f, middle) continuously monitors the battery level of the devices through Bluetooth communication and turns on the RF transmitter for wireless power transfer if the battery level goes below 15% (Supplementary Fig. 10). This closed-loop charging scheme prevents the battery from being fully discharged, thereby making the devices always on standby for wireless triggering. Figure 2g and Supplementary Movie 3 show a proof-of-principle demonstration of the automatic wireless charging of the devices. Real-time measurement of the battery voltage level of the devices during operation within a wireless charging cage verifies the capability of the wireless closed-loop system, which will not necessitate physical interference with freely moving animals during behavioral experiments.

### Mechanical and thermal characteristics of soft wireless optoelectronic systems

**Fig. 3:**
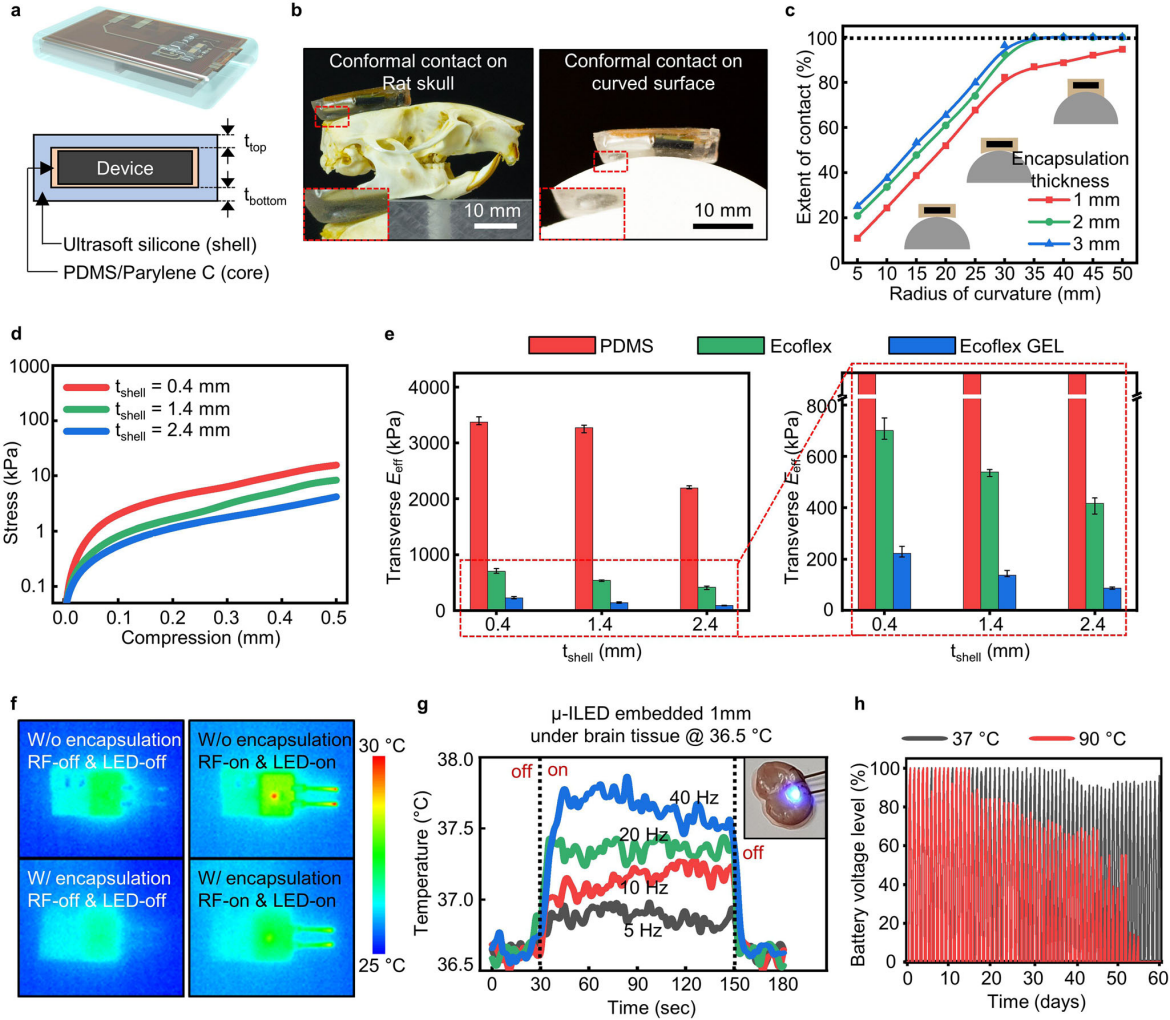
Mechanical and thermal characteristics of soft wireless rechargeable optoelectronic implants. **a** Schematic diagram of an implant encapsulated with soft biocompatible polymers (top) and its cross-sectional view (bottom). **b** Optical images of the implant conformally mounted on a rat skull (left) and a half-cylinder structure with a radius of curvature of 35 mm (right). The insets show the zoomed-in images of the device edge, highlighting perfect conformal integration with the curved surfaces. **c** Extent of conformal contact on half-cylinder structures with various radii of curvature (5–50 mm) for the devices with silicone gel encapsulation with different thicknesses (*t*_shell_ = 0.4, 1.4, and 2.4 mm). **d** Mechanical stress as a function of compression for devices coated with silicone gel with different thicknesses (*t*_shell_ = 0.4, 1.4, and 2.4 mm). **e** Transverse effective Young’s modulus (*E*_eff_) of devices coated with three common elastomers (PDMS, Ecoflex, and Ecoflex GEL) as a function of the encapsulation thickness (left) (*n* = 3). The zoomed-in graph (right) highlights the significantly low effective Young’s modulus of a device with Ecoflex GEL encapsulation, compared with devices coated with PDMS or Ecoflex. Error bars indicate maximum and minimum values. **f** Infrared images showing surface temperature of devices without (top) and with (bottom) polymer encapsulation before (left) and during (right) wireless charging and μ-ILED operation (40 Hz, 10 ms pulse width). The measurement was made in an ambient environment at room temperature. **g** Temperature of explanted brain tissue with a μ-ILED operated at different pulse frequencies (5, 10, 20, and 40 Hz; 10 ms pulse width) at 1 mm beneath the tissue surface (inset). To mimic a biological environment, the baseline temperature of the explanted brain tissue was maintained at 36.5 °C using a heater. **h** Battery voltage level as a function of time during repeated device operation—that is, repetition of wireless charging (60 min) and μ-ILED operating (20 Hz, 10 ms pulse width)—after immersing the devices in saline water with temperatures of 37 °C and 90 °C.

Wirelessly rechargeable optoelectronic systems are packaged into a soft, tissue-compatible device platform that can adapt to deformation and conform to curvilinear surfaces inside the body. The soft packaging platform consists of a thin bilayer (core) of PDMS (0.6 mm) and Parylene C (7 μm), which acts as a waterproof barrier against biofluid penetration, and an outer ultrasoft silicone gel (shell; Ecoflex GEL, Smooth-On Inc.; 33.4 kPa; 1.4 mm), which works as a mechanical buffer (Fig. 3a). This core/shell soft polymer composite system provides several key features for fully implantable systems; it offers (i) perfect conformal integration with curved body surfaces, (ii) thermomechanical compatibility between the implant and soft brain tissue, (iii) protection of the electronic system from biofluid, and (iv) light device weight that cannot be achieved using conventional encapsulation materials such as metals and glass. Figure 3b shows optical images of polymer-encapsulated devices (5 mm in total thickness) conformally interfaced with curved surfaces of a rat skull (left) and a hemispherical structure (right; the radius of curvature of 35 mm). Further, as shown in Fig. 3c and Supplementary Fig. 11, the extent of contact on the curved surface is enhanced with an increasing thickness of the outer ultrasoft silicone layer, saturating to peak values with a total encapsulation thickness of 2 mm (1.4 mm thick Ecoflex GEL shell and 0.6 mm-thick PDMS core). With this encapsulation, the devices can make perfect conformal integration with any surface with a radius of curvature of 35 mm or greater without bending the device structure inside the soft polymer coating, thus ensuring stable and consistent device performance (Supplementary Fig. 12). Besides, the stress–strain analysis revealed that the transverse effective Young’s modulus of the devices were substantially decreased to as low as ~137 kPa, which is comparable to the modulus of pure soft silicone (e.g., Dragon Skin, Smooth-On Inc.), when an ultrasoft silicone (Ecoflex GEL) with the thickness larger than 1.4 mm is used as the encapsulating shell (Fig. 3d, e and Supplementary Fig. 13). Our soft packaging with optimized parameters (i.e., Shell_top/bottom_ = 0.5 mm/0.9 mm, Core_top/bottom_ = 0.5 mm/0.1 mm) based on this mechanical analysis not only makes the overall device sufficiently thin for full implantation, but also ensures excellent conformity as well as biomechanical compatibility, all of which are desirable for subdermal implants.

In addition, the core/shell coating works as a thermal buffer as well as a fluid barrier that enables thermally safe and waterproof operation in a biofluid environment. The polymer encapsulation layers (2 mm for the device body; 7 μm Parylene C for μ-ILEDs) effectively dissipate the heat generated during wireless charging and μ-ILED operation, thus preventing brain tissue from being thermally damaged (Fig. 3f and g and Supplementary Fig. 14). The maximum temperature increase during device operation (when the μ-ILEDs are operated at 40 Hz with a 10 ms pulse width) is minimal (~1.1 °C), thus satisfying the standard for thermally safe operation of medical devices (i.e., maximum allowed temperature increment over the body temperature: <2 °C; ISO 14708-1:2014(E)^30^). In our durability test inside a saline solution (0.9%) at two different temperatures, 37 °C and 90 °C (Supplementary Fig. 15a), the polymer encapsulation provided excellent waterproofing, which allowed the devices to operate stably for at least 55 days at <90 °C (Fig. 3h and Supplementary Fig. 15b). According to the Arrhenius relationship^24,31^, the lifetime of the devices is estimated to be longer than a year at 37 °C, which demonstrates their potential usability for chronic in vivo studies.

### Control of the expression of cocaine-induced locomotor sensitization by wireless rechargeable optoelectronic systems in freely moving rats

**Fig. 4:**
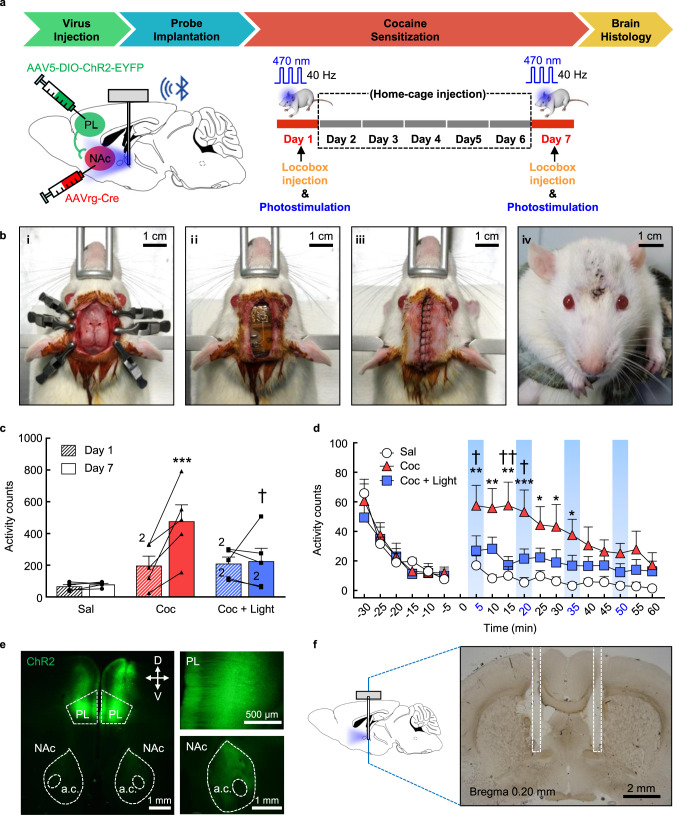
Control of the expression of cocaine-induced locomotor sensitization using wireless rechargeable optoelectronic implants in freely moving rats. **a** Calendar outlining the timeline for the whole experimental procedure. A schematic diagram of the rat brain shows locations for virus injections and μ-ILED probe insertion. The cocaine injection schedule is also illustrated (right). A sub-group of cocaine-injected rats was photostimulated at days 1 and 7. **b** A series of photos for a rat taken during the surgery for device implantation (i–iii) and after recovery from the surgery (iv). **c** Total locomotor activity counts observed during the 60 min test on days 1 and 7 after saline (white), cocaine only (red), or cocaine with photostimulation (blue) (*n* = 5). Two individual data scores overlapped are marked as number 2 next to them. Data were analyzed by two-way repeated ANOVA followed by post hoc Bonferroni comparisons. ***p < 0.001, cocaine only group on day 7 compared to day 1. ^†^*p* < 0.05, cocaine group with photostimulation compared with cocaine only group. Error bars indicate mean + SEM. **d** Time-course data at day 7, which are shown as locomotor activity counts with 5 min intervals obtained during the 30 min preceding (−30 through 0 min) and the 60 min following saline (white), cocaine only (red), or cocaine with photostimulation (blue) (0–60 min) (*n* = 5). The blue bar indicates light stimulation delivered (5, 20, 35, and 50 min). ***p < 0.001, **p < 0.01, *p < 0.05, cocaine only group compared to saline. ^††^*p* < 0.01, ^†^*p* < 0.05, cocaine only group compared with cocaine with photostimulation. Error bars indicate mean + SEM. **e** Epifluorescence image of a diagonal brain section with a low magnification (left), showing AAV-mediated EYFP expression in both the PL and NAc core areas. With a higher magnification (right), it is more evident that EYFP expressed well in cell body regions in the PL (upper right) and even at the axon terminal location in the NAc core (bottom right). **f** Representative bright-field image with probe tracks. Most tracks are found in the area behind the NAc.

In order to test whether our wireless optoelectronic device works functionally and controls behaviors effectively in freely moving animals, we conducted optogenetic experiments by adopting a well-known cocaine locomotor sensitization scheme, following adeno-associated virus (AAV)-mediated channelrhodopsin-2 (ChR2) expression in a prelimbic (PL) to nucleus accumbens (NAc) circuit and device implantation (Fig. 4a). For the implantation of the device, sufficient area of the skin above the rat skull was incised to fit the device (1.4 g; 19 × 12 × 5 mm^3^; Fig. 1c) and holes were drilled through the skull for probe injection (Fig. 4b, i). Once the probes were injected into brain tissue and the device body was mounted on the skull using a cyanoacrylate adhesive and dental cement, the opened skin was closed and sutured for full implantation of the device (Fig. 4b, ii–iii). After a week of recovery (Fig. 4b, iv), the rats were all healthy and showed natural normal behaviors (i.e., eating, moving, rearing, and grooming) without any notable deficits in activity and motor coordination, as shown in Supplementary Movies 4 and 5. Figure 4c shows the locomotor activity counts obtained during day 1 and 7 in response to intraperitoneal (IP) injection of saline, cocaine only, and cocaine with photostimulation into the NAc. As well-known^32,33^ and expected, rats exposed to daily cocaine showed a more profound sensitized locomotor response on day 7 compared with day 1 (*p* < 0.001). This effect, however, was significantly inhibited by concurrent photostimulation (470 nm wavelength, 40 Hz with 10 ms pulse width, 30 s on/off for 5 min with 10 min light-free interval) to the NAc core. The two-way repeated measures analysis of variance (ANOVA) conducted on these data indicates that there are multiple significant effects on locomotor activity of groups [F(2,12) = 5.75, *p* < 0.02], days [F(1,12) = 10.04, *p* < 0.009], and interactions between groups and days [F(2,12) = 7.54, *p* < 0.009]. Post hoc Bonferroni comparisons revealed that photostimulation significantly decreased (*p* < 0.05) the sensitized locomotor activity produced by cocaine for a total of 60 min. Time-course analyses for locomotor activity data obtained at day 7 (Fig. 4d) showed that the sensitized locomotor-activating-effects of cocaine persisted for approximately the first half hour of testing, and the ability of optogenetic stimulation to inhibit these effects was apparent throughout the course of this time. The ANOVA verified significant effects on locomotor activity of groups [F(2,12) = 6.94, *p* < 0.02] and time [F(11,132) = 4.64, *p* < 0.001]. Post hoc Bonferroni comparisons showed that photostimulation significantly decreased (*p* < 0.05–0.01) the sensitized locomotor activity caused by cocaine at the 5, 15, and 20 min time points compared with that observed in rats that received cocaine only. After the experiments, we verified ChR2 expression in both PL and NAc core regions where viruses were delivered (Fig. 4e) and accurate probe implantation in the area right below the bregma (Fig. 4f) where μ-ILED probes were optimally placed to illuminate the NAc region toward the anterior direction (see brain diagram in Fig. 4a). These results clearly indicate that our wireless device is fully implantable and functionally works well in freely moving animals, thus confirming its potential for in vivo optogenetics.

### Proof-of-concept demonstration for potential operation in human brains

**Fig. 5:**
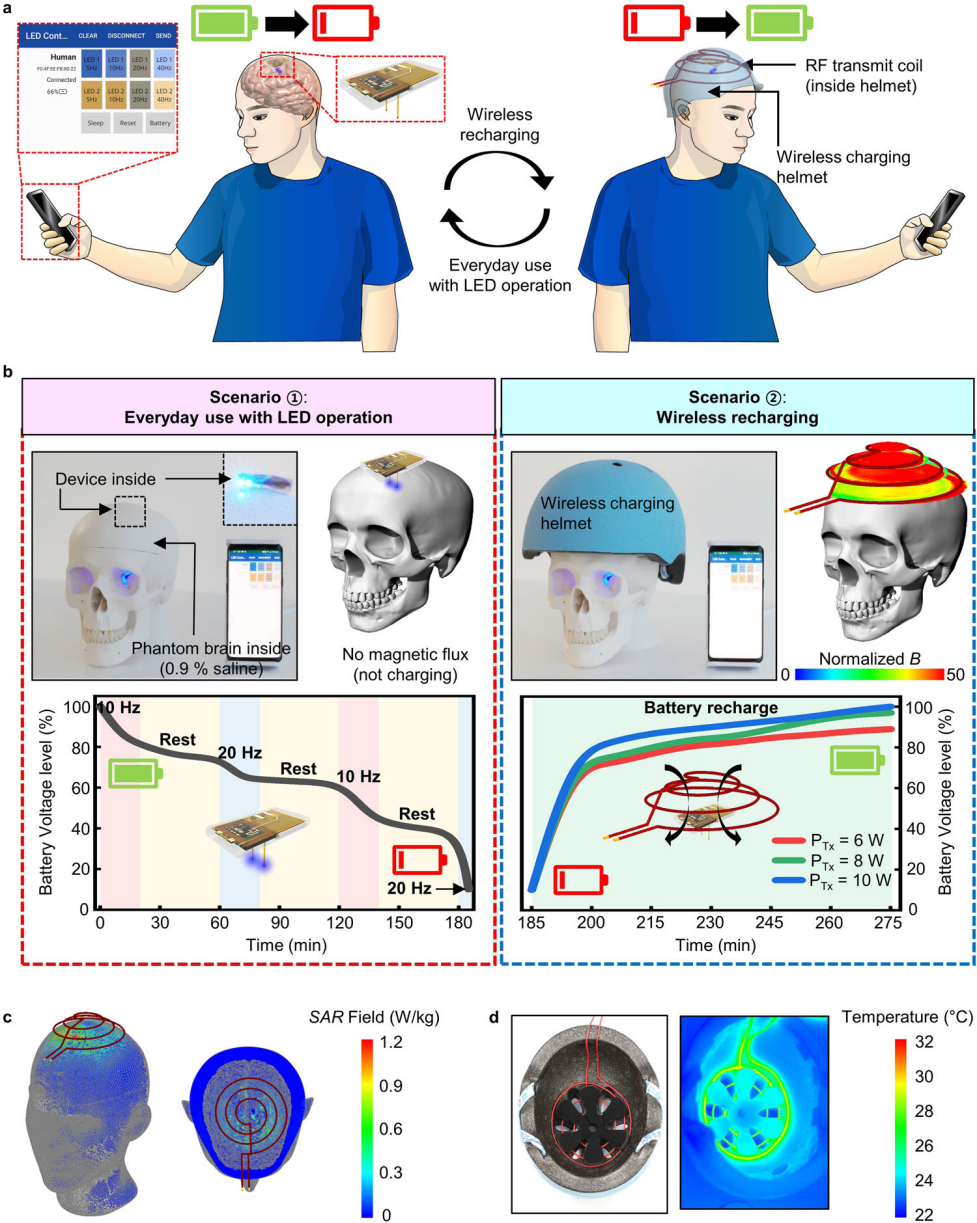
Proof-of-concept demonstration of fully implantable, wireless rechargeable optoelectronic systems for potential operation in a human brain. **a** Conceptual illustration showing wireless operation and recharging of the system for chronic human brain applications. A man with the wireless system implanted in his/her brain can operate it by simple manipulation of a smartphone (left) and recharge the battery by wearing a wireless charging helmet integrated with an RF transmit coil (right). **b** Optical images and electrical characteristics of the system, implanted in a model human head constructed with a phantom skull and brain (a balloon filled with 0.9% saline water), for two different operation scenarios: (1) everyday use with LED operation and (2) wireless recharging. To simulate everyday use (left), a set of μ-ILED operation (10 and 20 Hz, 10 ms pulse width; ~20 min) and rest (~30 min) was repeated until the device battery was almost fully discharged (battery level ~10%). The device was successfully charged by transmitting RF powers (6, 8, and 10 W) using a custom-designed wireless charging helmet (>70% charging in 15 min). **c**, **d** Simulated illustration of the specific absorption rate (SAR) over the human head **c** and IR image of the RF transmit coil **d** when a transmitting RF power of 10 W at 6.78 MHz is supplied to a wireless charging helmet. Sufficiently low SAR and negligible heat generation induced by the wireless charging helmet verifies biologically safe operation.

The proposed fully implantable, wirelessly rechargeable, soft optoelectronic system may open new opportunities for enabling optogenetics in the human brain for therapeutic applications. Figure 5 illustrates the proof-of-concept demonstration of such system for its potential operation in the human brain. The fully implantable wireless system (Fig. 5a) can be used as a user-friendly clinical device that can be controlled by a simple smartphone manipulation for optical stimulation of the target neural circuits (left) and that can be wirelessly recharged using a wireless charging helmet (right, Supplementary Fig. 16a). Empowered by the miniaturized, battery-integrated wireless architecture, this system not only requires minimalistic hardware (i.e., a smartphone) for its control, but also enables its ubiquitous operation for treatment of brain disorders, thereby making it a highly practical tool for use in daily life. This feature overcomes limitations of contemporary battery-free wireless optogenetic devices, which are restricted to use in animal studies but not humans, owing to the need for special, bulky wireless power transfer setup to turn on the devices^20–27^. Furthermore, with the wireless charging capability, the device does not require disruptive multiple surgeries to replace the battery, adding value as a patient-friendly, chronically implantable device.

Figure 5b shows experiments using a phantom skull (Classic Human Skull Model 3 part, 3B Scientific) and a phantom brain (a balloon filled with 0.9% saline solution, Supplementary Fig. 16b) to study the feasibility of device operation in the human brain. In this study, we successfully and reliably achieved both wireless LED controls (scenario 1) and wireless charging (scenario 2). Note that, for human applications, the device can integrate a larger size of battery, therefore, the device operation time can be substantially increased (Supplementary Fig. 9c), compared to the one measured in scenario 1 in Fig. 5b. Compared with a rat cage with loop antennas (Fig. 2b, f), the size difference between the transmitter (in a helmet) and receiver coils (in the implant) was relatively small, producing a denser magnetic field and thus enabling faster wireless charging. Moreover, this wearable charging helmet approach facilitates efficient wireless power transfer since the implant always stays at the same location relative to the transmitting helmet. According to our simulation (Fig. 5c and Supplementary Fig. 17), wireless charging with relatively low RF input power (<10 W) leads to a small specific absorption rate (SAR < 1.2 W/kg), which satisfies the Federal Communications Commission (FCC) guideline for biologically safe operation (SAR of 1.6 W/kg; FCC 1.1310^34^). In our experiment simulating the wireless charging (Supplementary Fig. 18), this small SAR actually resulted in negligible temperature increase in phantom brain tissue (<0.1 °C), verifying the RF safety of our system. In addition, heat generation from the wireless charging helmet during input power supply was also negligible (maximum temperature in the antenna structure ~31.0 °C in an ambient environment), thus ensuring its thermally safe operation (Fig. 5d and Supplementary Fig. 19). All these features make this system an attractive option for potential applications to the human brain for treatment of neurological or psychiatric disorders such as Parkinson’s disease^35–39^.

## Discussion

We presented a fully implantable, wirelessly rechargeable, and smartphone-controlled optogenetic system that combines the advantages of both battery-powered (i.e., a stable power supply) and battery-free devices (i.e., a miniaturized design), which allow reliable, ubiquitous operation and seamless full implantation in the body, respectively. This device harnesses not only the ability for simultaneous and selective wireless control of multiple animals using a readily available smartphone, but also closed-loop wireless auto-charging capability to enable intervention-less, long-term in vivo studies. These features, integrated within a minimalistic and biocompatible platform, can accelerate neuroscience research through rapid setup and powerful wireless control in any in vivo environment, thus contributing to exploration of brain functions and treatment of various neurodegenerative diseases.

Despite the novel features of this device, its design can be further improved. Although the dimensions (1140 mm^2^) are ~55% smaller and the weight (1.4 g) is ~42% lighter than our previous head-mounted neural implant^19^, which thus allows subdermal implantation in rats, the current proposed design does not allow full integration into mice, primarily owing to the bulkiness of a Bluetooth SoC module and the rechargeable battery. Using a bare minimum BLE SoC (e.g., SmartBond TINY™ DA14531, Dialog Semiconductor) and ultrathin flexible batteries (e.g., Polymer Matrix Electrolyte batteries, BrightVolt) will further scale down the device size. With integration of these miniaturized state-of-the-art electronic components, future devices will be able to both facilitate its seamless surgical implantation into animals as small as mice and reduce post-surgical stress in the animals. Furthermore, although the proposed device is compatible with X-ray and computed tomography (Fig. 1e and Supplementary Fig. 4), it is not compatible with magnetic resonance imaging (MRI) yet, primarily because of the presence of magnetically susceptible metal interconnects and a battery, which restricts its potential for broad clinical applications. Substituting these specific parts with non-ferromagnetic components will ensure safety for real-time MRI studies while the device is implanted^40–42^. Likewise, although the core/shell soft polymer coating structure can enable stable operation of the device within the body over a year, more advanced hermetic sealing is required to substantially increase the lifetime of the implantable devices. Thermally grown silicon dioxide is considered a semi-permanent biofluid barriers^43,44^. Adoption of this material for the device encapsulation can improve the lifetime of the device to several decades as demonstrated in the previous studies^43^. In addition, development and integration of multifunctional flexible probes^17,19,45–47^ with high-density, high-rate neural recording interfaces^48,49^, and long-life flexible lithium battery^50,51^ will open innovative opportunities for chronic, closed-loop neuromodulation by enabling continuous monitoring and precise analyses of convoluted neural activities as well as feedback-based optogenetic stimulations using a programmed algorithm.

With these improvements, we believe that the presented technology will help realize applications of optogenetics in humans in the near future, allowing highly precise target-specific treatment of neurodegenerative diseases such as Alzheimer’s and Parkinson’s^35–39^. Unlike conventional implantable devices, most of which require a surgical process to replace exhausted batteries after several years^52,53^, using wireless recharging for an implanted device can eliminate surgical stress for its chronic operation. Therefore, this technology can bring beneficial impacts for various implantable devices including deep brain stimulators^54^, cardiac pacemakers^55^, and gastric pacemakers^56^. Likewise, powerful customizable controls using a readily available smartphone will significantly facilitate therapeutic interventions. The device design introduced in this paper represents a generic implantable device platform with straightforward extensibility. Integration of advanced functions such as drug delivery and electrophysiological recording in this soft, fully implantable platform will further enhance its versatility and utility, broadening opportunities for in vivo neuroscience and clinical applications.

## Methods

### Fabrication of fully implantable, wirelessly rechargeable optoelectronic device

Four individual constituent layers of a fully implantable, wireless rechargeable optogenetic device were fabricated through a photolithography process. Copper traces were patterned on a PI substrate (25 μm thick) at each layer. The patterned copper traces on the top and bottom layers (both 18 μm thick) provide the pathways for signaling between the surface-mounted electronic components, whereas the copper traces in the middle two layers (both 35 μm thick) work as a wireless power-scavenging coil antenna in the device, which results in inductance of ~3.2 μH. A Bluetooth Low Energy System-on-Chip (BLE SoC) and other electronic components were mounted on the copper electrodes of a flexible substrate (25 μm thick) after a low-temperature solder paste (T5, SMDLTLFP10T5, Chip Quik) was applied. They were soldered in a reflow oven (AS-5060, SMTmax) at a peak temperature of 215 °C for 90 s. μ-ILED probes were fabricated similarly on a 25 μm-thick flexible PI substrate coated with a 35 μm thick copper layer on each side (total 130 μm-thick, 300 μm-wide, and 17.5 mm-long). Probes were then assembled along their length on the flexible circuit after μ-ILEDs (470 nm, TR2227, Cree) were attached on their tips. Afterward, the device was encapsulated with PDMS (0.6 mm; Sylgard 184, Dow Corning), Parylene C (7 μm), and Ecoflex GEL (1.4 mm; Smooth-On). 3D-printed (3DP-310F, CUBICON) molds were used to encapsulate the device with PDMS and Ecoflex GEL.

### RF transmitter for wireless energy transfer

An RF transmitter system consists of an RF signal generator, an RF power amplifier, a heat sink fan, a DC power supply, three sets of loop antenna with a resonant frequency matching circuit board, and a custom-made closed-loop automatic electrical switch. It wirelessly transfers the alternating current to the implanted loop antenna, which in turn supplies the harvested energy to the rechargeable battery in the device. In order to amplify the transmitter power, the output of the RF signal generator (Model 3390, Keithley) was connected to the input of the RF power amplifier (1061-BBM1C3FEL, Empower RF Systems) via SMA to BNC cable. A DC power supply (DP30-05TP, TOYOTECH) was used to supply the DC voltage (26-30 V) to the RF power amplifier and the heat sink fan (LA 21/200 24 V, Fischer Elektronik), which is necessary for cooling the heat generated from the amplifier. The frequency of the RF signal was set to 6.78 MHz, and the power amplitude was set to 0 dBm at the RF signal generator. Each set of the loop antenna was constructed with a transmitter coil and resonant frequency matching capacitors, which were connected in series. Output of the RF power amplifier was linked to each terminal of three sets of loop antennas to supply an amplified RF signal. The other side of the terminals of the loop antennas were combined and connected to the custom-made electrical switch, which automatically controls the current flow to the system based on the battery charge level; that is, it supplies the current when the battery charge level is <15%, and stops the current supply when the battery charge level reaches 100%.

### Design of wireless energy harvester for battery charging

The wireless energy harvester of the device consists of a two-layer coil antenna, a voltage-doubler circuit with a Schottky diode for preventing reverse-flow of the current, and a rechargeable LiPo battery (GMB-300910, PowerStream Technology; 12 mAh, 9 mm × 10 mm × 3 mm, 0.3 g). A 100 pF capacitor (GRM0335C1E101JA01J, Murata Electronics; 0.6 mm × 0.3 mm × 0.3 mm) was connected in parallel with a coil antenna with an inductance of ~3.2 μH to match the resonant frequency of 6.78 MHz. Wirelessly received signal was rectified and multiplied through a voltage-doubler circuit, which was composed of three Schottky diodes (PMEG4002AESFYL, Nexperia USA Inc.; 0.6 mm × 0.3 mm × 0.3 mm) and two charge pump capacitors (GRM155R60J475ME47D, Murata Electronics; 4.7 μF, 1.0 mm × 0.5 mm × 0.7 mm). The stabilized signal was supplied directly to the LiPo battery for charging, while the RF transmitter system was turned on. The wirelessly charged LiPo battery supplies the output DC voltage to the BLE SoC (RFD77101, RF Digital Corporation) through a linear voltage regulator (NCP4624DMU30TCG, ON Semiconductor; 1.0 mm × 1.0 mm × 0.6 mm) and two decoupling capacitors (GRM033R61A104KE15D, Murata Electronics; 0.1 μF, 0.6 mm × 0.3 mm × 0.3 mm).

### Electromagnetic simulation of field generation

Commercial software ANSYS HFSS (ANSYS HFSS 19) was used to simulate the magnetic field density around the loop antennas and the specific absorption rate (SAR) to a human’s head. The loop antennas used in the rat cage and the wireless charging helmet were modeled using the same software. Magnetic fields were plotted at different spatial heights inside the rat cage model and the wireless charging helmet model in order to show the overall magnetic field distribution visually. The human’s head model for SAR simulation was imported from the 3D component library of ANSYS HFSS, which reflected frequency dependent material properties (i.e., relative permittivity ~581.1 and electrical conductivity ~0.234 s/m).

### Measurement of compression–stress curves

The compression–stress curves for the encapsulated devices were measured using a digital force gauge (M5-100, Mark-10). The devices were encapsulated in nine different combinations of three materials (PDMS, Ecoflex, and Ecoflex GEL) and three thicknesses (0.4, 1.4, and 2.4 mm) (Supplementary Fig. 11a). An applied force with increasing compression was measured and recorded in real time through MESUR Lite software while the load of the force gauge compressed the device. The stress owing to compression was calculated by dividing the applied force by the device area.

### Measurement of temperature change in brain tissue during μ-ILED operation

The temperature increment of rat brain tissue during μ-ILED operation was measured to verify its biothermal compatibility. μ-ILED probes were embedded under a slice of explanted rat brain (1 mm thick). In order to mimic an in vivo environment, the measurement was conducted on a hot plate (MSH-50D, DAIHAN-brand), in which the temperature was maintained at 36.5 °C, after immersing the rat brain tissue in saline water (0.9%). The temperature of the rat brain surface was measured using a longwave infrared thermal camera (A655sc, FLIR) during μ-ILED operation. μ-ILEDs were operated for a period of 2 min at different frequencies (5, 10, 20, and 40 Hz with 10 ms pulse width) using a custom smartphone app.

### Animal subjects

Male Sprague–Dawley rats weighing 280–310 g (9 weeks old) on arrival were obtained from Orient Bio Inc. (Seongnam-si, Korea). They were housed two per cage in a 12-hr light/dark cycle room (lights out at 8:00 pm), and all experiments were conducted during the day time. Rats had access to food and water ad libitum at all times. All animal use procedures were conducted according to an approved Institutional Animal Care and Use Committee protocol of Yonsei University College of Medicine.

### Stereotaxic surgery

One week after arrival, the rats were anesthetized with IP ketamine (100 mg/kg) and xylazine (6 mg/kg), and placed in a stereotaxic instrument with the incisor bar at 5.0 mm above the interaural line. A pair of bilateral infusion cannulas (28 gauge; Plastics One, Roanoke, VA) connected to 1 μL syringes (Hamilton, Reno, NV) via PE-20 tubing were angled at 10° to the vertical and aimed at the NAc core (A/P, +3.2; L, ±2.8; D/V, −7.1 mm from the bregma and skull)^57^ delivering retrograde AAV expressing Cre recombinase (AAVrg-hSyn-Cre, viral titer 7 × 10¹² vg/mL) (Addgene, Watertown, MA; the Addgene plasmid #105553 used to prepare this virus was a gift from Dr. James M. Wilson) and at the PL (A/P +3.2, L ±1.3, D/V −4.1 mm from the bregma and skull) delivering Cre-dependent AAV expressing ChR2-EYFP or EYFP alone (AAV5-EF1α-DIO-hCHR2-(H134R)-EYFP or AAV5-EF1α-DIO-EYFP, viral titer 1 × 10¹³ vg/mL) (Addgene, Watertown, MA; the Addgene plasmids #20298 and #27056 used to prepare these viruses were gifts from Dr. Karl Deisseroth). In all, 1 μL syringes were placed on an infusion pump (KD Scientific, Holliston, MA) and the virus was infused at a rate of 0.1 μL/min for 5 min with an additional 5 min allowed for its diffusion. The incised skin covering the skull was fixed with surgical staplers and the rats were returned to their home cages for recovery and virus expression. After two weeks, the rats were re-anesthetized with IP ketamine (100 mg/kg) and xylazine (6 mg/kg), and placed in a stereotaxic instrument with the incisor bar not raised. A wirelessly rechargeable optoelectronic device was placed directly onto the rat skull and bilateral μ-ILED probes were vertically inserted aiming at the far posterior site of the NAc right below the bregma (A/P +0.5, L ±2.0, D/V −7.5 mm from the bregma and skull)^58^. The device was helped to adhere to the skull with a cyanoacrylate adhesive (Henkel, Dusseldorf, Germany), and a dental adhesive resin cement (Sun Medical, Shiga, Japan). The incised skin was closed with non-absorbable sutures with additional help of a topical skin adhesive (Ethicon, Somerville, MA) and the rats were returned to their home cages for one week of recovery.

### Drugs

Cocaine hydrochloride was purchased from Belgopia (Louvain-La-Neuve, Belgium). It was dissolved in sterile 0.9% saline to a final concentration of 15 mg/mL.

### Locomotor activity

Locomotor activity was measured with a bank of nine activity boxes (35 × 25 × 40 cm^3^) (IWOO Scientific Corporation, Seoul, Korea) made of translucent Plexiglas. Each box was individually housed in a polyvinyl chloride plastic sound-attenuating cubicle. The floor of each box consisted of 21 stainless steel rods (5 mm diameter) spaced 1.2 cm apart center-to-center. Two infrared light photobeams (Med Associates, St. Albans, VT), positioned 4.5 cm above the floor and spaced evenly along the longitudinal axis of the box, estimated horizontal locomotor activity. Locomotor activity was counted only when two beams were consecutively interrupted. In this way, any confounding measures like grooming in a spot covering just a single beam was avoided from the counts.

### Cocaine sensitization

About 1 week after probe implantation surgery, rats were randomly assigned to three groups. Rats in one group were administered with saline once daily and the ones in the other two groups with cocaine (15 mg/kg, IP) for 7 days. Locomotor activities after saline injection, or cocaine with or without optical stimulation, were measured in activity boxes only on days 1 and 7, whereas no activities were measured during home cage-injections for the other days. In this way, administering cocaine in different places (i.e., in the activity boxes for the first and the last injections and in home cages for the other injections) helped to avoid any confounding effects of conditioning development. This commonly used procedure is well known to produce enduring sensitization of the locomotor response to cocaine^32,33^.

One day prior to day 1, in order for the rats to adjust to the new procedure and environment, all the rats were first put in plastic restrainers for 30 min and then placed in activity boxes for another 30 min. They were all administered saline and returned to the activity boxes for an additional 60 min. On days 1 and 7, all the rats were put in plastic restrainers for 30 min, and only a sub-group of cocaine (cocaine + optical stimulation) was wirelessly charged during this time, followed by habituation for 30 min in activity boxes. Then, the rats were administered saline, or cocaine with or without optical stimulation, and their locomotor activities were measured for 60 min. The implanted optoelectronic devices were paired with a smartphone through Bluetooth and operated using a customized smartphone app. A sub-group of cocaine with optical stimulation received repeated blue lights (470 nm) for 5 min (40 Hz, 30 s on/off) with an interval of a light-free period for 10 min throughout a total of 60 min locomotor activity measurement.

### Tissue preparation and histology

After completion of the behavioral experiments, the rats were deeply anesthetized with ketamine (100 mg/kg) and xylazine (6 mg/kg) and then perfused transcardially with 10 mM phosphate-buffered saline (PBS, pH 7.4) followed by 4% paraformaldehyde (PFA) solution in 10 mM PBS (pH 7.4). Their brains were removed and further post-fixed in 4% PFA at 4 °C for 24 h, followed by keeping them in a 30% sucrose solution for 72 h. Then, the brains were embedded in an O.C.T. compound (Tissue-Tek, Torrance, CA), frozen on dry ice, and stored at −80 °C. One set of coronal sections (100 μm) were made from the anterior portion, including the target areas for virus injections of frozen brain tissues, and their captured fluorescent images were analyzed for ChR2-EYFP expression. Another set of coronal sections (50 μm) were made from the surrounding area, including target areas for probe implantation of frozen brain tissues, and their bright-field images were analyzed for a μ-ILED probe track with reference to a stereotaxic atlas. The sections were all cover-slipped with a Vectashield mounting medium (Vector Laboratories, UK) before analysis, and all images were captured with a DP71 digital microscope camera attached to a fluorescent microscope (Olympus, Japan).

### In vivo biocompatibility studies

Twelve rats were anesthetized with IP ketamine (100 mg/kg) and xylazine (6 mg/kg) for device implantation. Two types of device encapsulation were used for comparison: one is PDMS alone (a well-known biocompatible silicone material (USP class VI) and the other is Ecoflex GEL surrounding the PDMS/Parylene C bilayer coating, and each of them was implanted subdermally under the rat scalp. The device was adhered to the skull with cyanoacrylate adhesive (Henkel, Dusseldorf, Germany) and the incised skin was closed with non-absorbable sutures. On day 3, 6, 10 after the surgery, the rats were anesthetized and hair on the scalp around the surgery site was removed by a depilatory cream (Nair, Ewing, NJ). Then, rats were perfused transcardially with 10 mM phosphate-buffered saline (PBS, pH 7.4) followed by 3.7% formaldehyde solution. The scalp tissue right above the device was excised out as a rectangle (5 × 10 mm) and post-fixed in 3.7% formaldehyde for 24 h. The tissue samples were embedded in paraffin and cut with 4 μm sections. The sections were then stained with hematoxylin and eosin (H & E). Digital images (×10 magnification) were obtained using DP71 digital microscope camera attached to a microscope (Olympus, Japan). Six representative areas were taken per rat and analyzed using Image J for the thickness of loose areolar tissue, which is the sub-area of the scalp directly contacted with the device.

### Statistical analysis

Statistical analyses were performed using the Sigma Plot version 12.0 (Systat Software, San Jose, CA). The locomotor activity counts were analyzed with two-way repeated measures ANOVA, followed by Bonferroni post hoc comparisons. Differences between experimental conditions were considered statistically significant when *p* < 0.05.

### Reporting summary

Further information on the research design is available in the Nature Research Reporting Summary linked to this article.

## Supplementary information

Supplementary Information

Supplementary Movie 1

Supplementary Movie 2

Supplementary Movie 3

Supplementary Movie 4

Supplementary Movie 5

Reporting Summary

## Data Availability

The data that support the plots within this paper and other findings of this study are available from the corresponding author upon reasonable request.

## Code availability

The software code used for wireless optoelectronic systems is available from the corresponding author upon reasonable request.
