# Supplementary material for: Soft subdermal implant capable of wireless battery charging and programmable controls for applications in optogenetics
Source: Nat Commun. 2021 Jan 22;12:535. doi: 10.1038/s41467-020-20803-y (PMC7822865; doi:10.1038/s41467-020-20803-y)
Supplement: Supplementary file 1 — Supplementary Information [file 41467_2020_20803_MOESM1_ESM.pdf]

Supplementary Information for

Soft Subdermal Implant Capable of Wireless Battery Charging and Programmable Controls for Applications in Optogenetics

Choong Yeon Kim[1†], Min Jeong Ku[2†], Raza Qazi[1,3], Hong Jae Nam[1], Jong Woo Park[2], Kum Seok Nam[1], Shane Oh[1], Inho Kang[1], Jae-Hyung Jang[4], Wha Young Kim[2], Jeong-Hoon Kim[2*], Jae-Woong Jeong[1*]

†These authors contributed equally to this work.

*To whom correspondence should be addressed. E-mail: jjeong1@kaist.ac.kr (J.-W.J.); jkim1@yuhs.ac (J.-H.K.)

This supplementary file includes:

Supplementary Table 1
Supplementary Figures 1-19

Other supplementary material for this manuscript includes the following:

Supplementary Movies 1-5

Supplementary Table

Supplementary Table 1. Comparison of various state-of-the-art devices for wireless optogenetic systems[19,23,24,26]

Control Scheme	Wirelessly Rechargeable Implantable Optogenetics (This Work)	Programmable Head-mountable Optofluidics[19] (Qazi et al., 2019)	Programmable High Frequency (HF) Implantable Optogenetics[26] (Gutruf et al., 2018)	High Frequency (HF) Implantable Optogenetics[24] (Shin et al., 2017)	Ultra High Frequency (UHF) Implantable Optogenetics[23] (Park et al., 2016)
Fully implantable?	**Yes**	No	Yes	Yes	Yes
Operation (Power supply & Control)	**Wireless battery charging (6.78 MHz) & Bluetooth control**	Battery powered (Battery replacement required) & Bluetooth control	HF broadcasting (13.56 MHz)	HF broadcasting (13.56 MHz)	UHF broadcasting (2.3 / 2.7 / 3.2 GHz)
Operation Range	10 – 100 m (Omnidirectional control)	10 – 100 m (Omnidirectional control)	~0.3 m (Angular dependency)	~0.3 m (Angular dependency)	~0.3 m (Angular dependency)
Modalities	Optical	Optical & Fluidic	Optical	Optical	Optical
# of control channels within a device	2 (can increase up to 27)	6	4	1	3
Ubiquitous operation?	**Yes** (Battery powered)	Yes (Battery powered)	No (Power setup dependent)	No (Power setup dependent)	No (Power setup dependent)
Intervention free?	**Yes**	No (Battery and drug cartridge replacement required)	Yes	Yes	Yes
Programmable operation	**Yes**	Yes	Yes	No	No
Mechanical property of the device	**Ultrasoft** (Coated with soft silicone elastomer, Device's Modulus ~137 kPa)	Soft probe (Rigid outer casing of the device body)	Moderate (Partially coated with PDMS)	Moderate (Coated with PDMS)	Soft (Coated with PDMS)
Wireless re-programmability	Yes (Over the Air Update)	Yes (Over the Air Update)	No	No	No
Device selectivity within a large congested group	**Yes** (Software based wireless pairing using Smartphone)	Yes (Software based wireless pairing using Smartphone)	No, Limited (All devices receiving RF signal will be activated)	No, Limited (All devices receiving RF signal will be activated)	No, Limited (All devices receiving RF signal will be activated)
Channel selectivity without re-programming	**Yes** (Software based control using Smartphone)	Yes (Software based control using Smartphone)	No (Channels activated as beforehand programmed when receiving RF signal)	No (Single channel)	Yes (All channels matched at different resonant frequencies)
Closed-loop control	**Yes**	Yes	Yes	No	No

* Bold texts highlight the strengths of our work.

Supplementary Figures

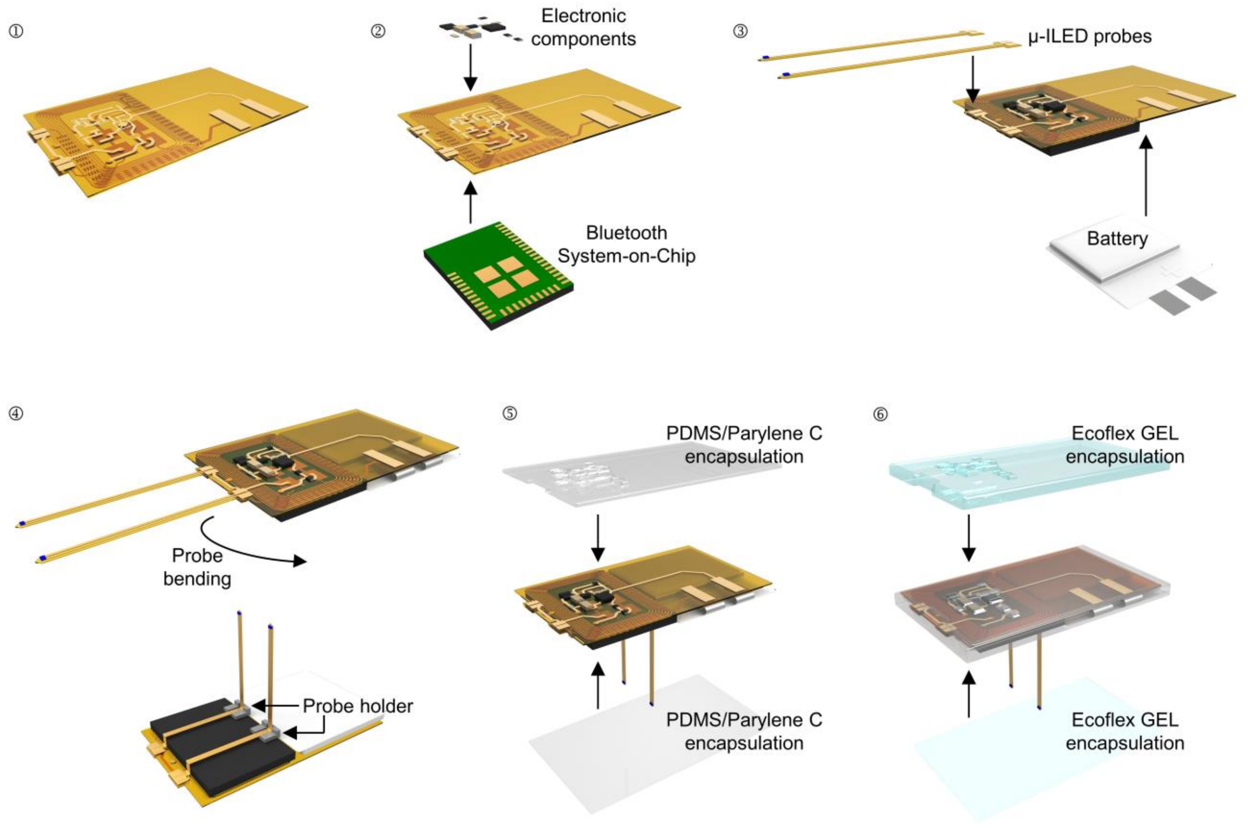

Supplementary Fig. 1 | Fabrication process of a fully implantable, wirelessly rechargeable, soft optoelectronic device. ① Fabricate a flexible circuit substrate consisting of four layers of copper traces on a separate polyimide layer through the standard photolithography process. ② Solder electronic components and Bluetooth Low Energy System-on-Chip on copper electrodes of the flexible substrate using solder paste. ③ Attach μ-ILED probes and a rechargeable Lithium Polymer battery on electrodes through direct soldering. ④ Bend the probes and fix them using 3D-printed probe holders. ⑤-⑥ Encapsulate the device with PDMS (0.6 mm), Parylene C (7 μm) (⑤), and Ecoflex GEL (1.4 mm; ⑥) using 3D-printed molds.

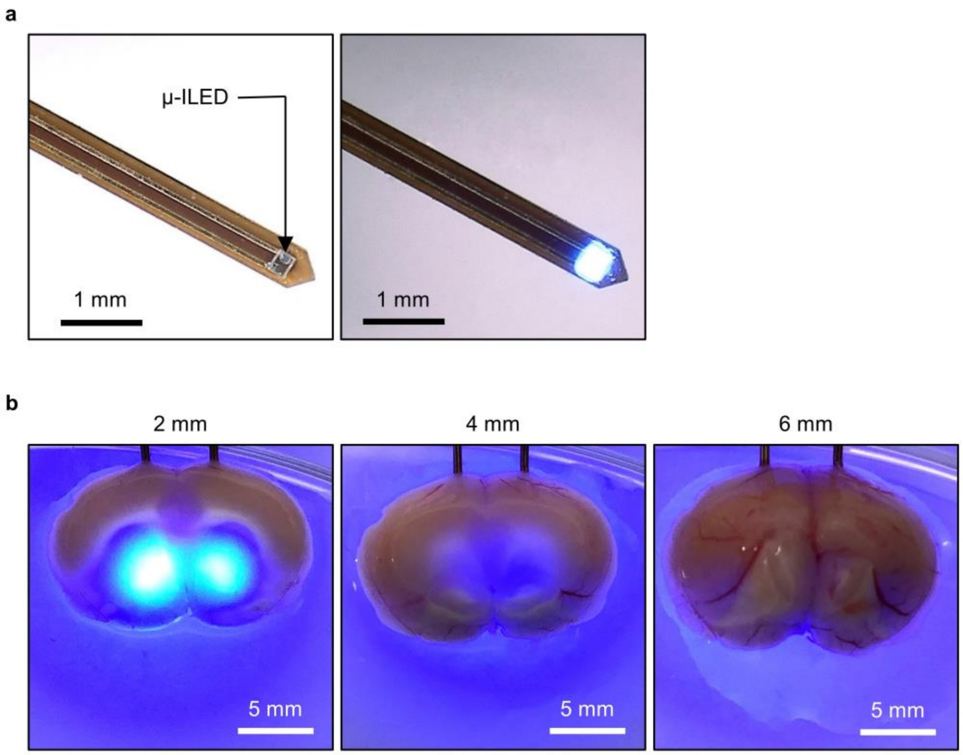

Supplementary Fig. 2 | Optoelectronic neural probe. a, Optical images of a flexible probe with a μ-ILED (blue, 470 nm wavelength) in off (left) and on (right) states. **b**, Optical images of working μ-ILED placed beneath the rat brain slices with different thicknesses of 2 (left), 4 (middle), and 6 mm (right).

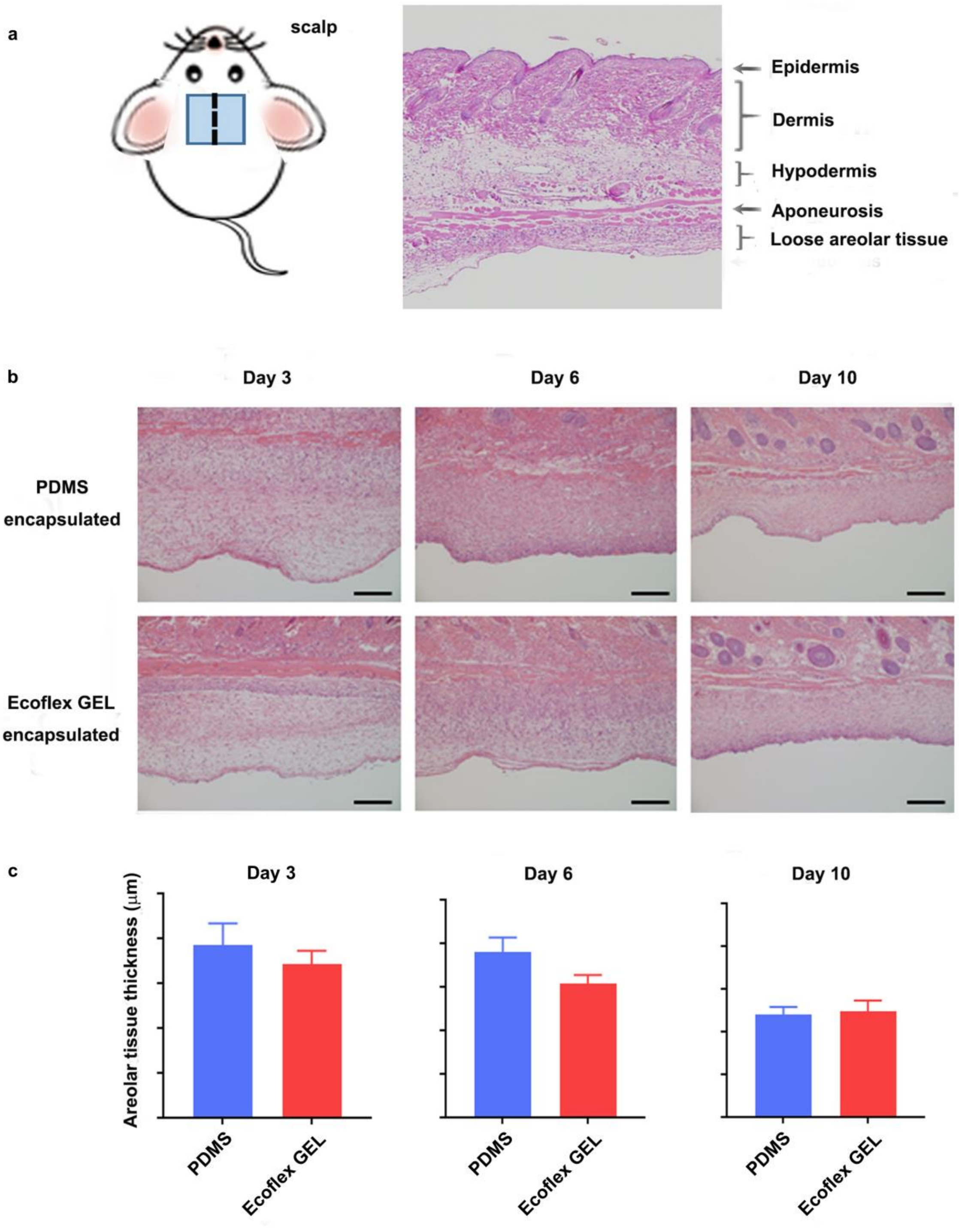

Supplementary Fig. 3 | Biocompatibility test for the fully implantable optoelectronic device with soft polymer encapsulation (outermost layer: Ecoflex GEL, inner layers: PDMS/Parylene C). a, Schematic diagram of a rat showing where tissues were taken

(left) and an image showing representative skin tissue stained with H & E with indications of each sub-area. **b,** Representative H & E stained skin tissue images obtained at 3, 6, and 10 days after surgery with PDMS- (control; USP class VI) and Ecoflex GEL-encapsulated devices implanted sub-dermally under the scalp. Scale bar is 200 µm. **c,** Analysis of loose areolar tissue thicknesses, indicating there are no significant differences between two groups (n = 12). Error bars indicate mean + SEM.

a

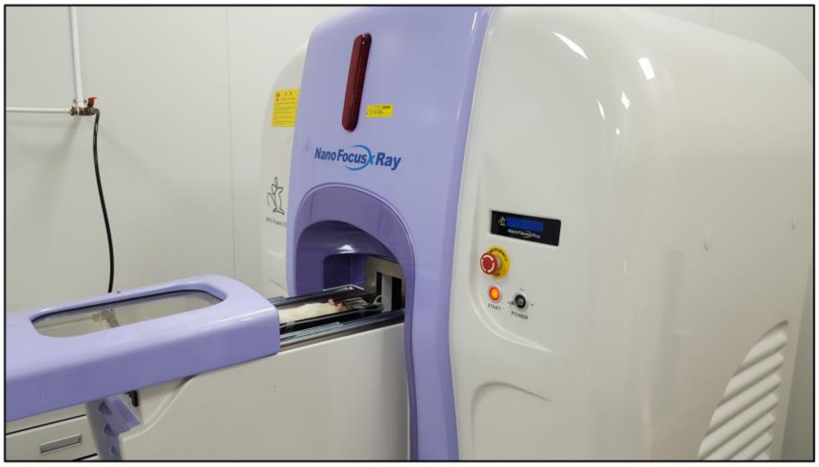

b

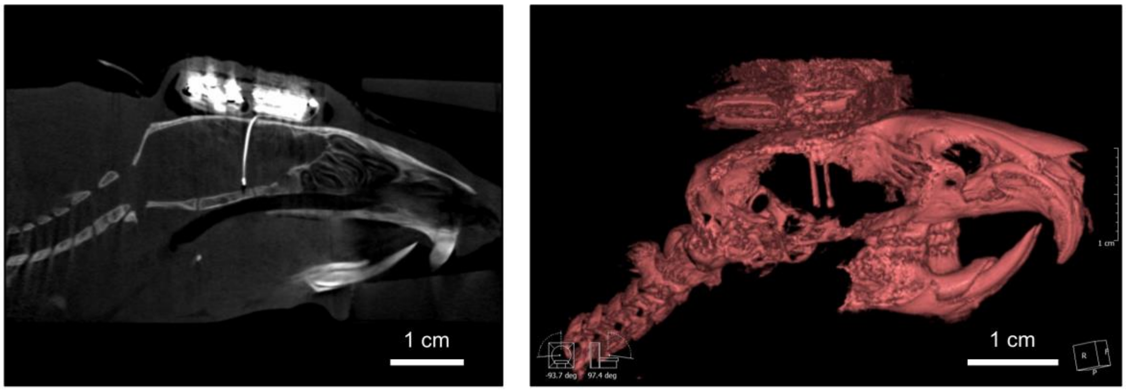

Supplementary Fig. 4 | CT imaging of a rat brain with a fully implanted wireless optoelectronic device. a, In order to view a real-time image of the rat brain during scanning, a rat was located inside a micro-CT scanner (NFR Polaris-G90) after implantation of wireless optoelectronic system. **b**, 2D (left) and 3D volume rendered (right) CT images of a rat implanted with a wireless optogenetic device.

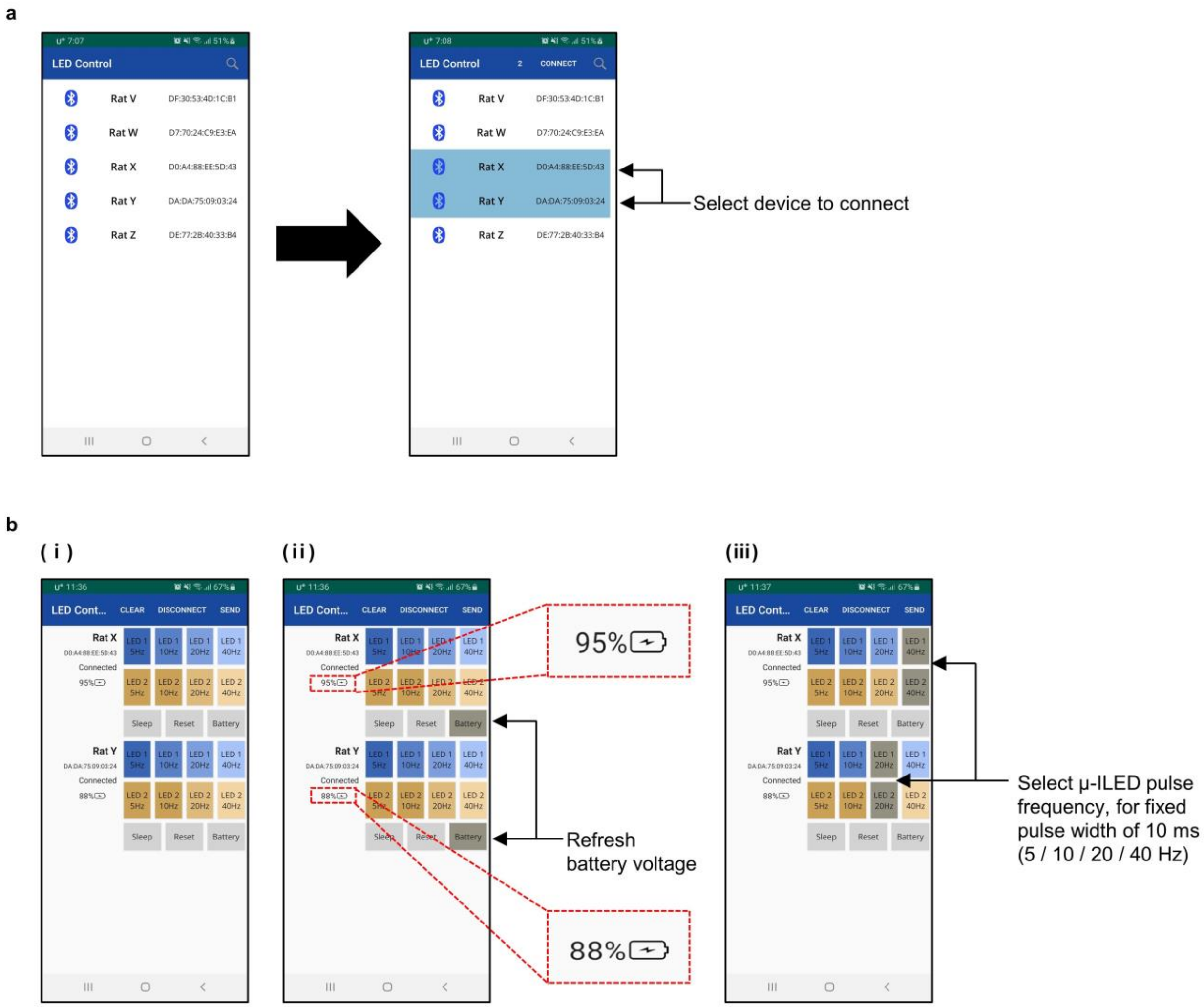

Supplementary Fig. 5 | Smartphone app user interface. a, Screenshot image of the device list page. This page displays all available BLE devices in the vicinity of the smartphone (left). From the list, multiple devices can be selected for simultaneous connection (right). **b**, Screenshot image of the LED control page (i). Battery voltage level of the paired BLE devices can be monitored wirelessly (ii), and operation conditions of μ-ILEDs (i.e. operation frequency and specific μ-ILED(s) to operate if multiple μ-ILEDs are integrated) can be selected (iii).

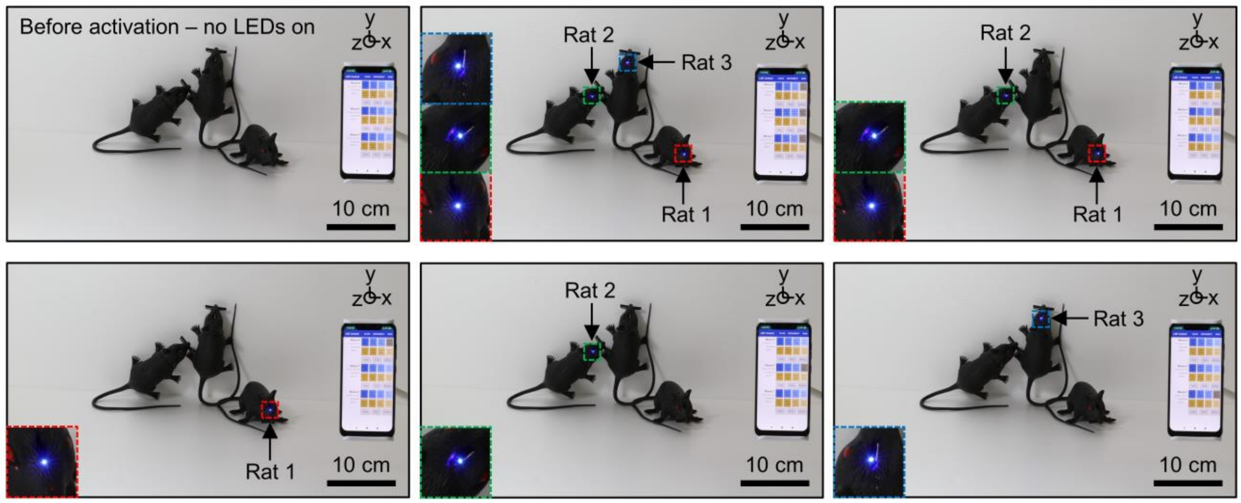

Supplementary Fig. 6 | Selective and orientation-independent control of wireless optoelectronic implants using a custom smartphone app. Smartphone Bluetooth control enables selective and angular orientation independent manipulation of wireless optogenetic devices implanted in model rats (each named Rat 1, 2, and 3) without any Line-of-Sight limitation that is prevalent with most contemporary wireless RF technologies.

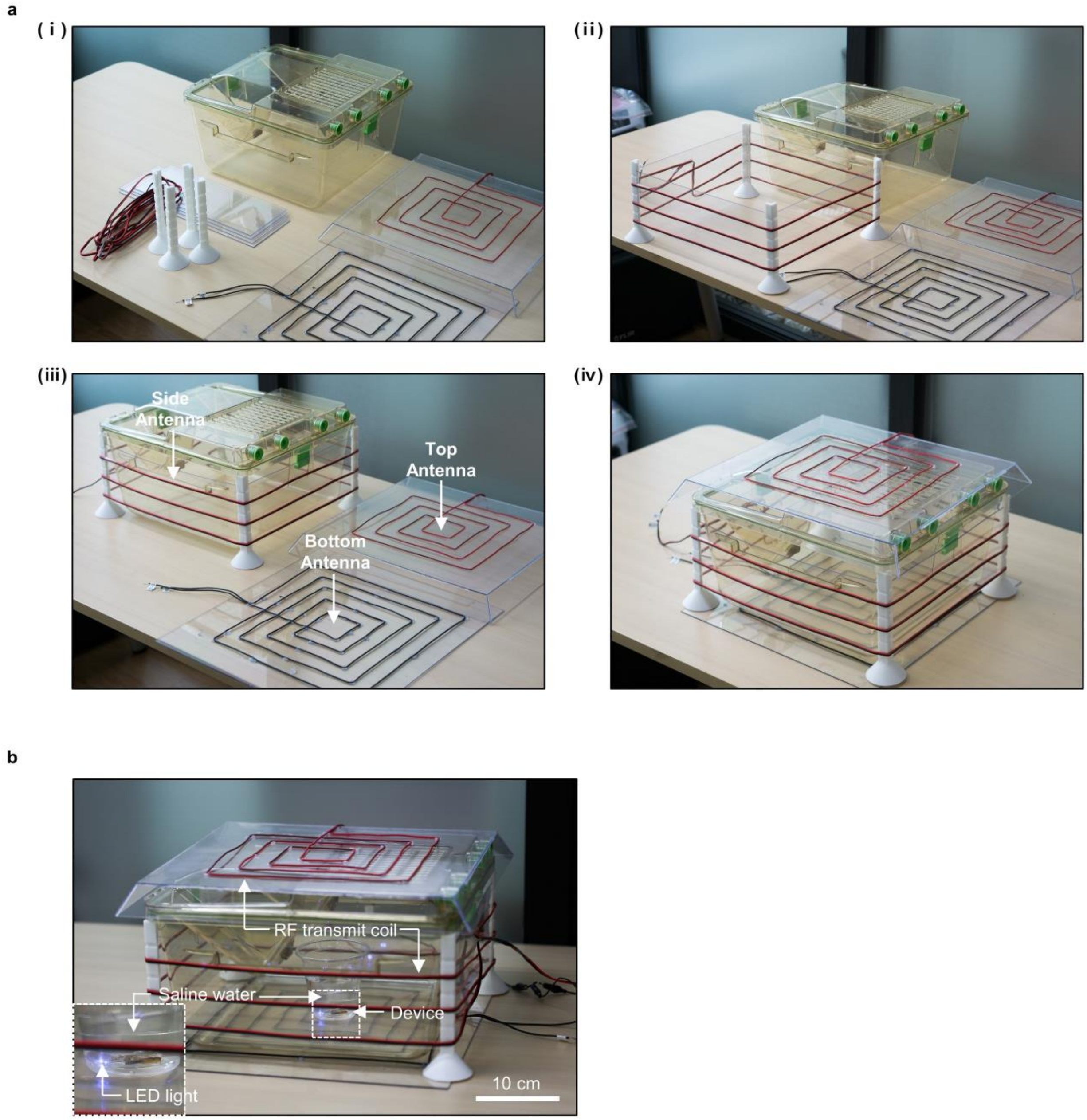

Supplementary Fig. 7 | Design of loop antennas integrated with a rat cage for wireless charging. a, Procedure of assembly of loop antennas (top, side, and bottom antennas) with a rat cage. **b**, Optical image of a rat cage with loop antennas, wirelessly charging an optoelectronic device immersed in saline water.

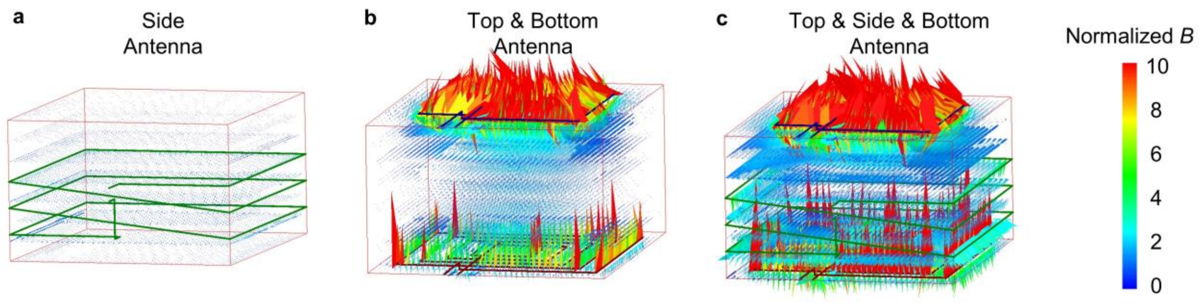

Supplementary Fig. 8 | Simulation showing normalized magnetic field distribution in a rat cage produced by different configurations of loop antennas. a-c, Magnetic field spatial dispersion when using the side loop antenna (**a**), the top and bottom antennas (**b**), and all of the three loop antennas (top, side, and bottom antennas) (**c**).

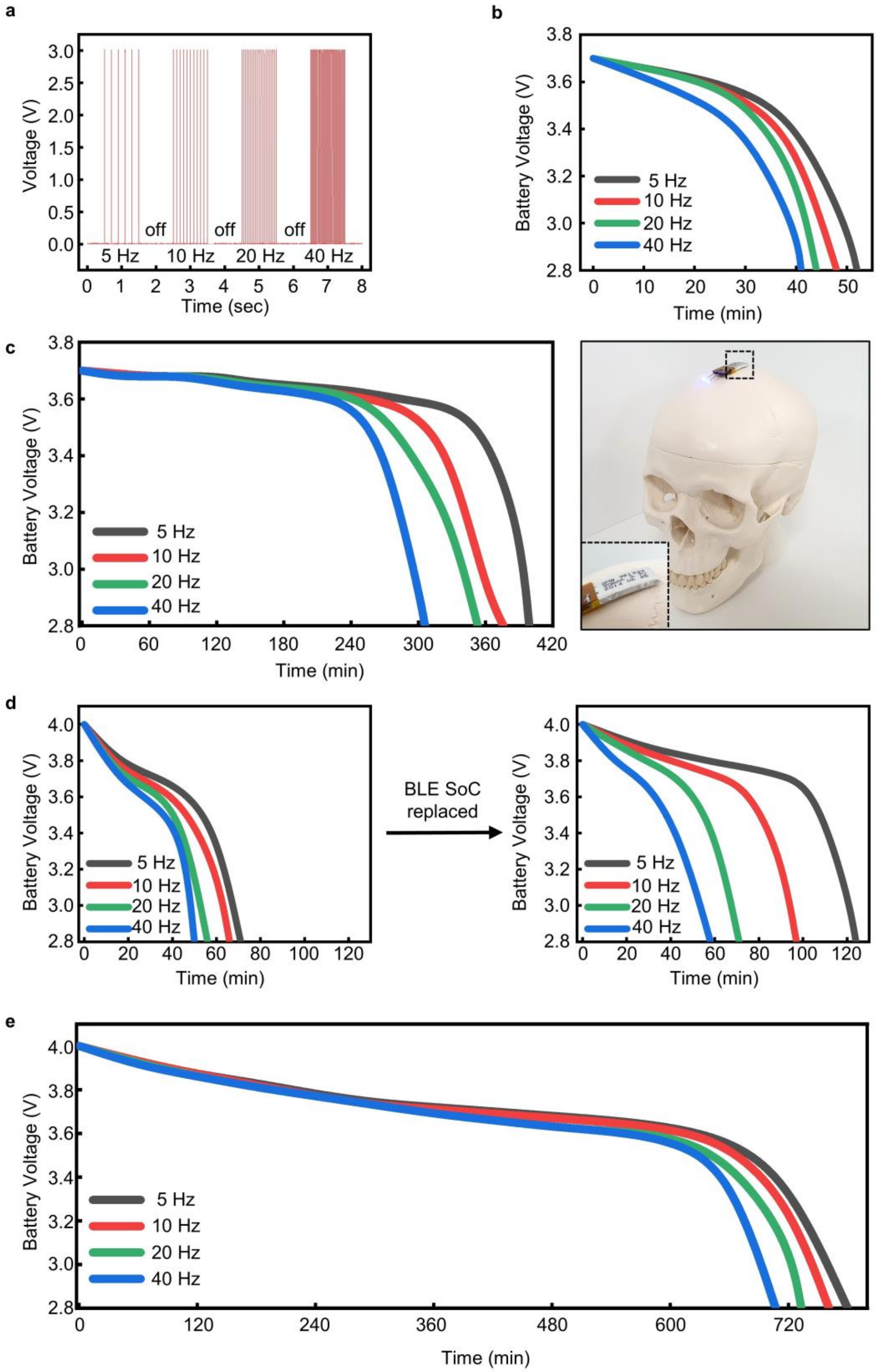

a
Voltage (V)
3.0
2.5
2.0
1.5
1.0
0.5
0.0
off
off
off
5 Hz
10 Hz
20 Hz
40 Hz
0 1 2 3 4 5 6 7 8
Time (sec)
b
Battery Voltage (V)
3.8
3.6
3.4
3.2
3.0
2.8
5 Hz
10 Hz
20 Hz
40 Hz
0 10 20 30 40 50
Time (min)
c
Battery Voltage (V)
3.8
3.6
3.4
3.2
3.0
2.8
5 Hz
10 Hz
20 Hz
40 Hz
0 60 120 180 240 300 360 420
Time (min)
d
Battery Voltage (V)
4.0
3.8
3.6
3.4
3.2
3.0
2.8
5 Hz
10 Hz
20 Hz
40 Hz
0 20 40 60 80 100 120
Time (min)
BLE SoC
replaced
Battery Voltage (V)
4.0
3.8
3.6
3.4
3.2
3.0
2.8
5 Hz
10 Hz
20 Hz
40 Hz
0 20 40 60 80 100 120
Time (min)
e
Battery Voltage (V)
4.0
3.8
3.6
3.4
3.2
3.0
2.8
5 Hz
10 Hz
20 Hz
40 Hz
0 120 240 360 480 600 720
Time (min)

Supplementary Fig. 9 | Electrical characteristics associated with μ-ILED operation. **a**, Measurement of μ-ILED voltage as a function of time. **b**, Battery discharging characteristics during μ-ILED operation at different pulse frequencies (5, 10, 20, and 40 Hz) with a 10 ms pulse width. Battery was discharged starting from 3.7 V, which is the nominal voltage of the lithium polymer (LiPo) battery. **c-e**, Battery discharging behaviors when integrating a flexible larger-capacity battery (90 mAh) (**c**), a state-of-the-art low power BLE SoC (**d**), and both of them (**e**), demonstrating the potential for significantly increased operation time. The μ-ILEDs were operated at different pulse frequencies (5, 10, 20, and 40 Hz) with a 10 ms pulse width.

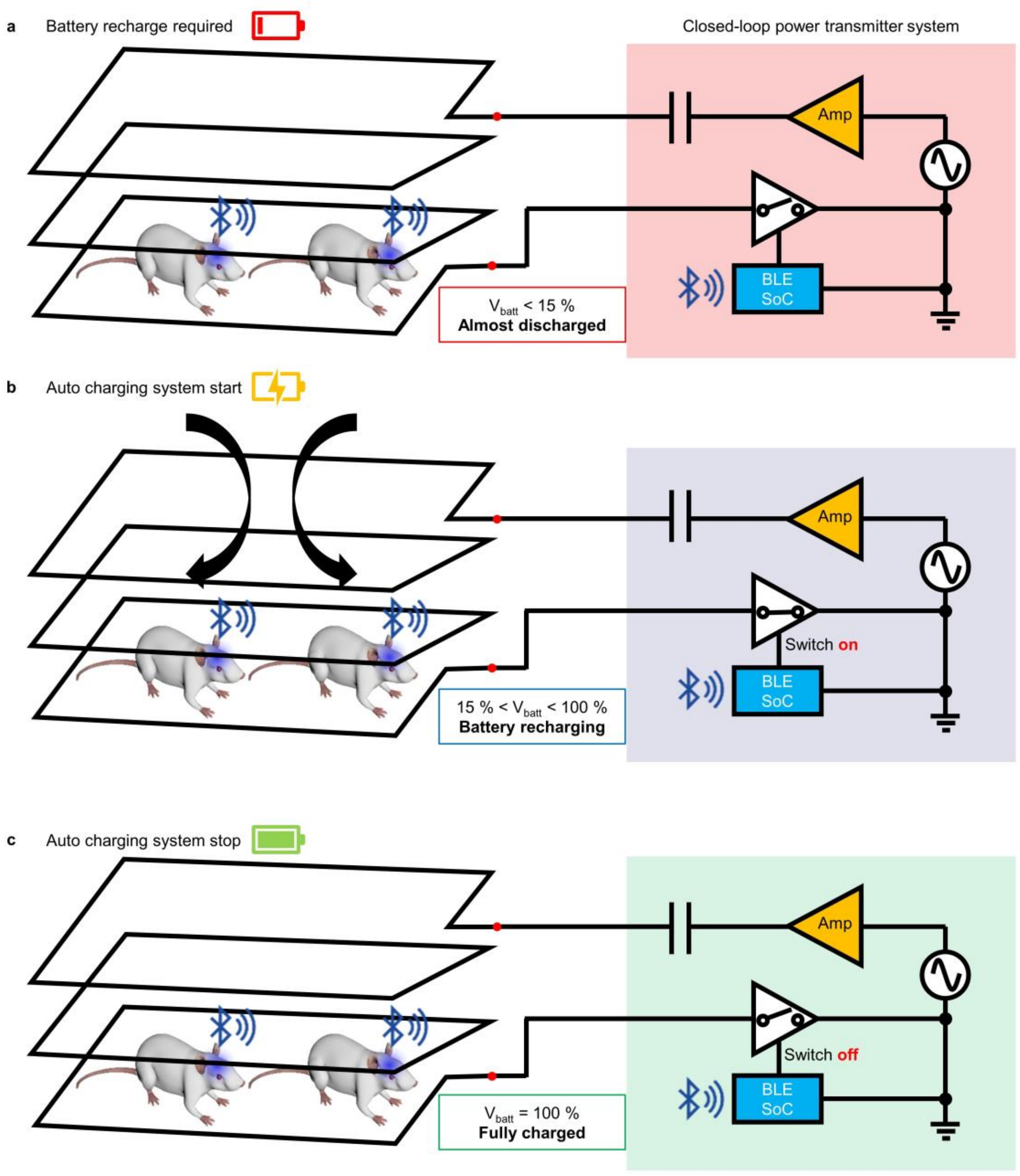

Supplementary Fig. 10 | Schematic diagram illustrating operation of the closed-loop auto-charging system. a, When the battery is almost discharged (battery voltage level < 15%), the wireless device implanted in a rat sends a trigger signal to the power transmitter system. **b**, The power transmitter system turns on to charge the battery. **c**, The wireless device sends another trigger signal to the power transmitter when the battery is fully charged (battery voltage level ~100%) before it stops the charging system.

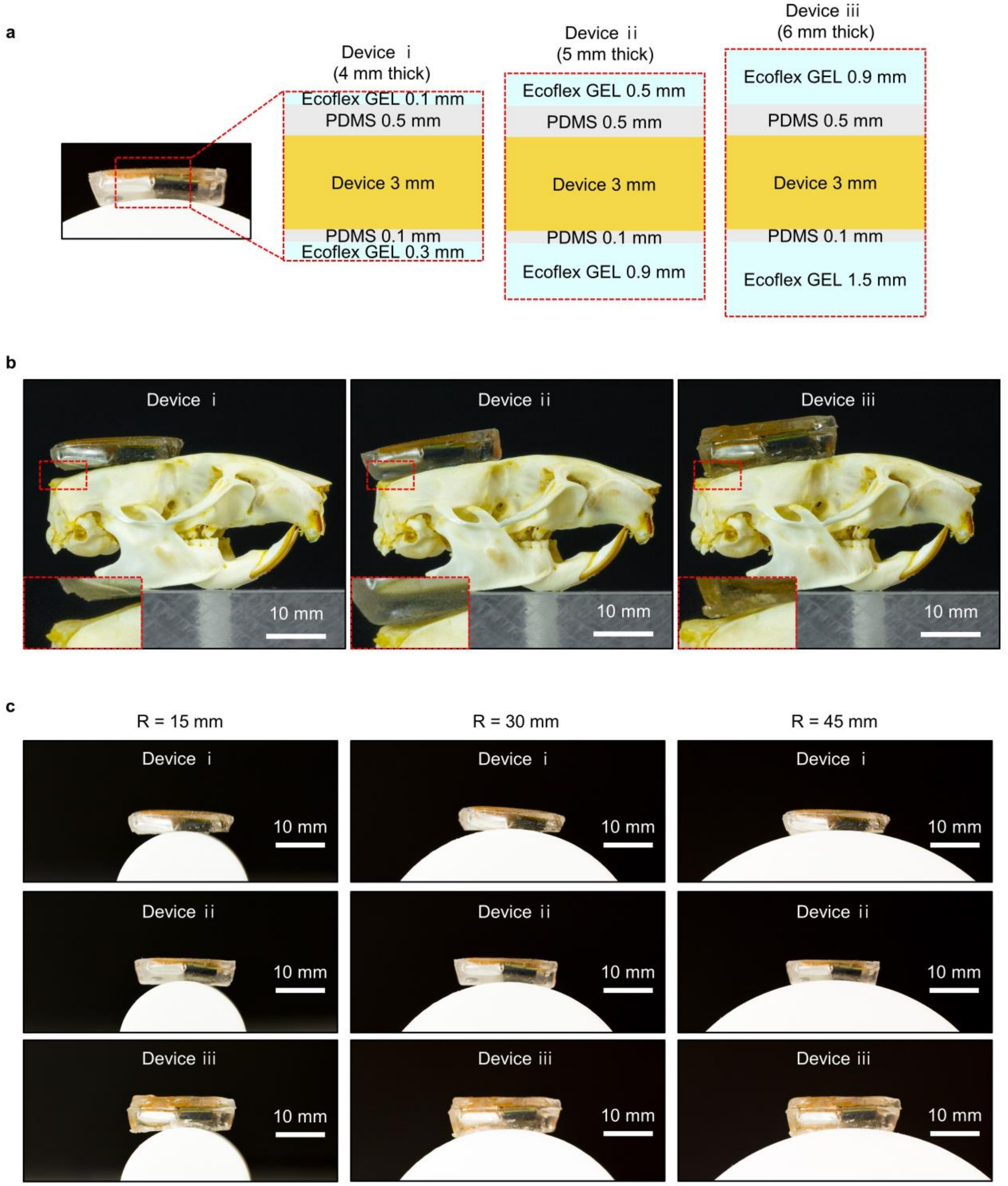

Supplementary Fig. 11 | Polymeric encapsulations and associated conformity of soft optoelectronic systems. a, Schematic illustration showing constituting materials and thickness of each layer for the devices with the total thickness of 4, 5, and 6 mm (t_{shell}

= 0.4, 1.4, and 2.4 mm, each). **b**, Optical images showing contact between the devices (i, ii, and iii) and a rat skull. The insets show zoomed-in images of the interface between the device edge and the skull. Devices ii and iii show perfect conformal contact on the rat skull unlike device i. **c**, Optical images showing contact between the devices (i, ii, and iii) and half-cylinder structures with different radiuses of curvature (15, 30, and 45 mm).

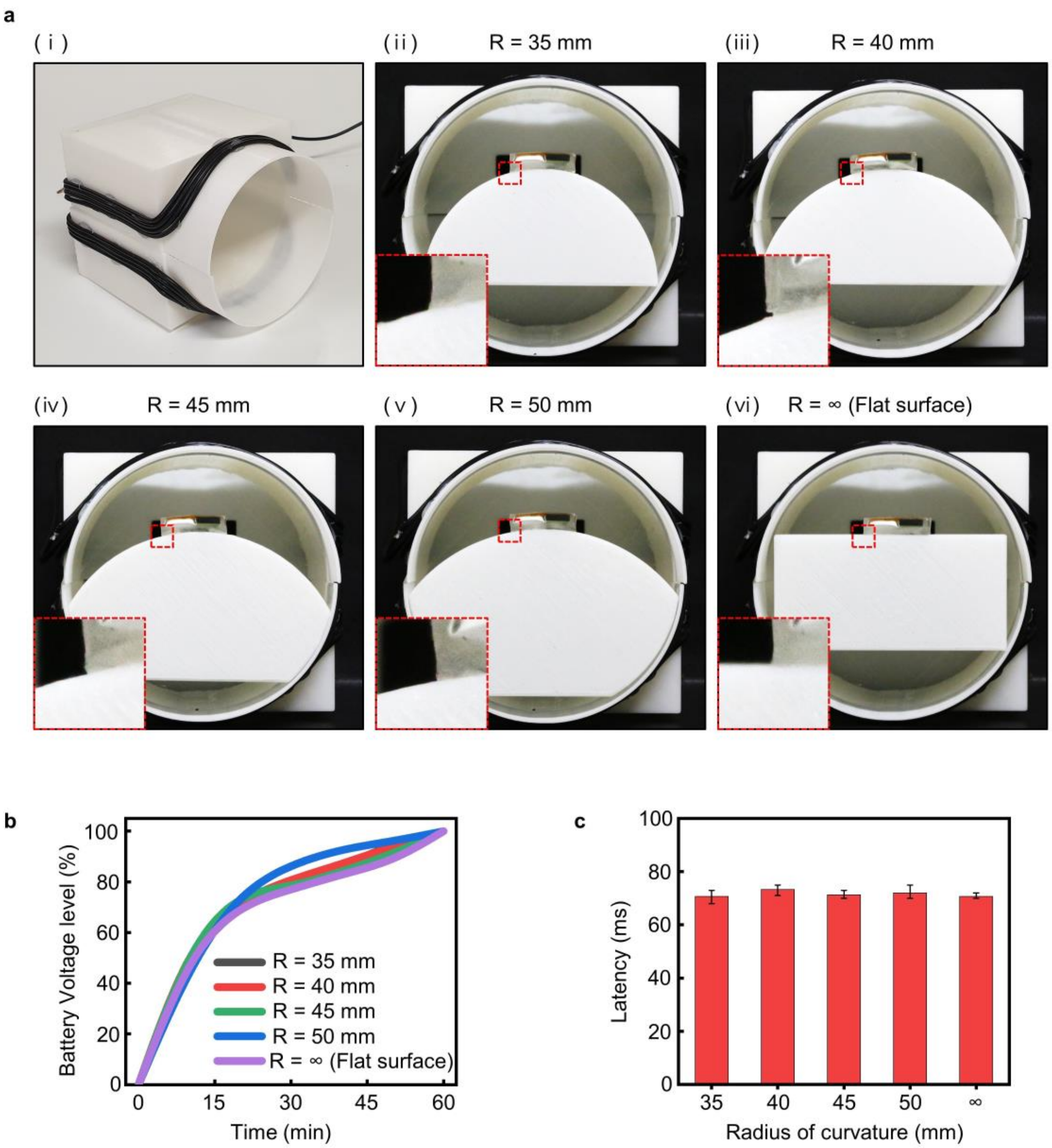

Supplementary Fig. 12 | Electrical characteristics of a device conformally interfaced with curved surfaces. a, Optical images showing a hollow cylinder structure surrounded with loop antennas (i) and contact between the device and half-cylinder structures with different radii of curvature (35, 40, 45, 50 mm, and ∞; ii-vi). The insets show magnified images of the interface between the device edge and the contact surface, emphasizing its perfect conformal contact. **b-c**, Wireless battery charging characteristics (**b**) and latency (n = 3) (**c**) of the device conformally interfaced with curved surfaces with

various radiuses of curvature (35, 40, 45, 50 mm, and ∞). These electrical characteristics verify stable and consistent device operation, which are not influenced by the quality of conformal contact. Error bars indicate maximum and minimum values.

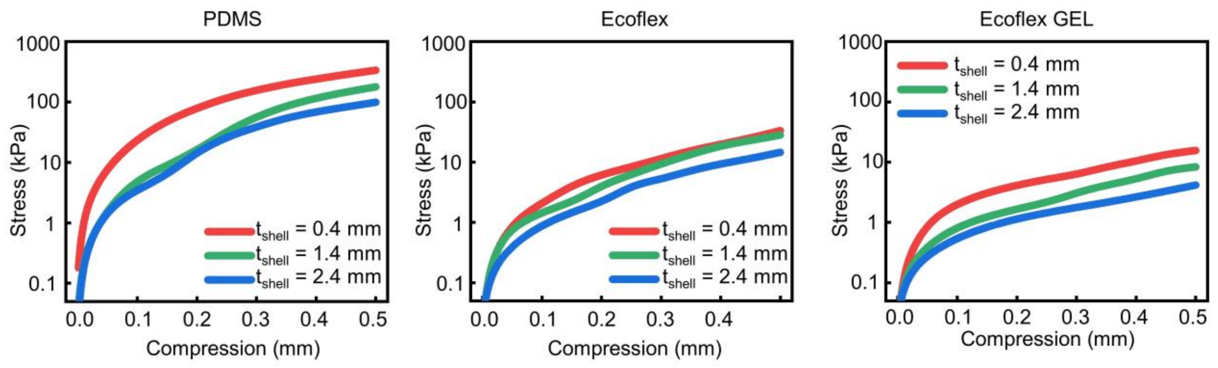

Supplementary Fig. 13 | Stress-compression curves of soft optoelectronic systems. Mechanical stress as a function of compression for devices coated with different elastomers (PDMS, Ecoflex, and Ecoflex GEL) with various thicknesses (t_{shell} = 0.4, 1.4, and 2.4 mm).

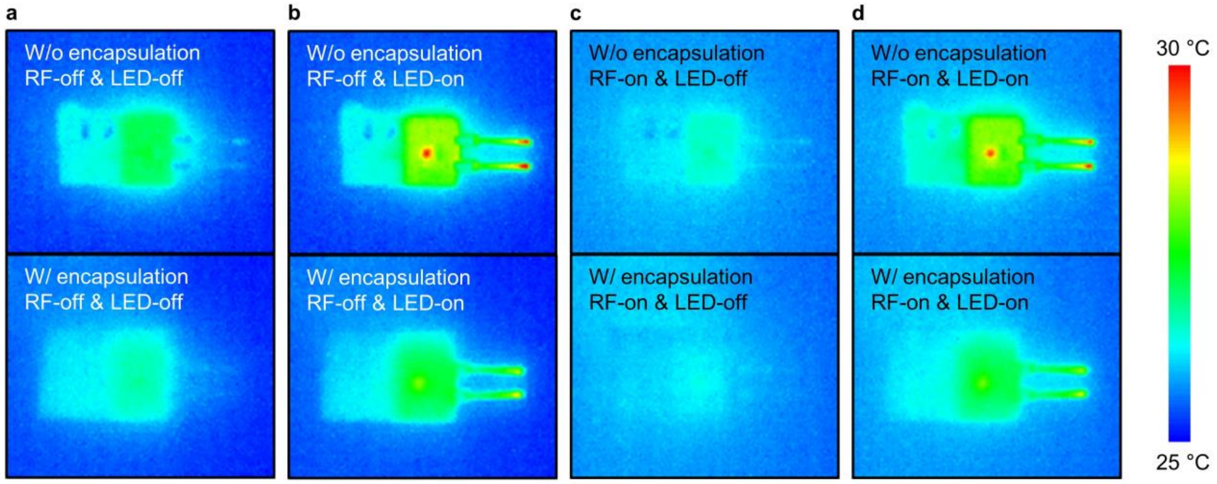

Supplementary Fig. 14 | Infrared images showing surface temperature of devices without (top) and with (bottom) polymer encapsulation for four different operation scenarios. a-d, Both RF and LED off (**a**), RF off and LED on (**b**), RF on and LED off (**c**), Both RF and LED on (**d**). The measurement was made in ambient environment at room temperature.

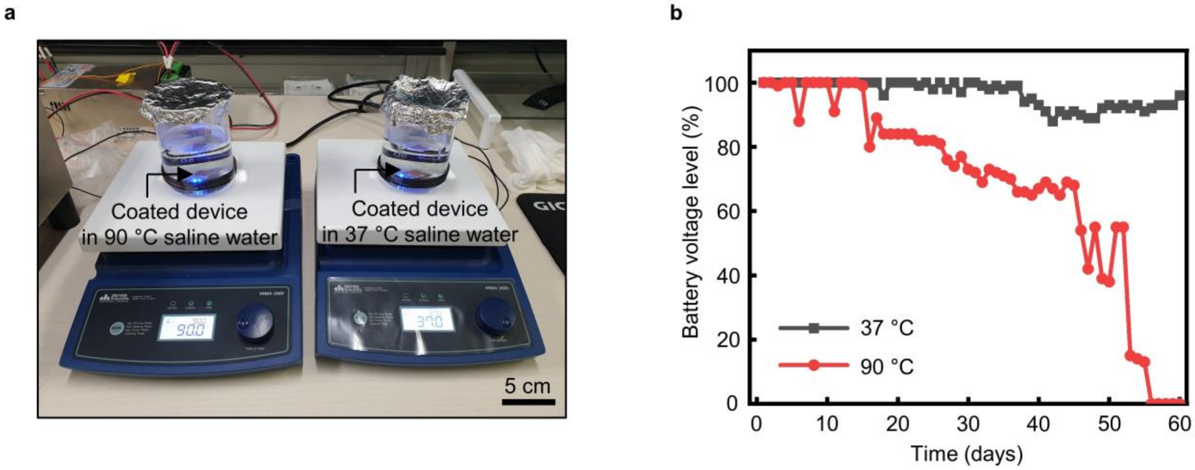

Supplementary Fig. 15 | Experimental setup for testing device reliability and durability in saline water with elevated temperature. a, For testing, devices were immersed in beakers with saline water maintained at 90 °C and 37 °C. The beakers, which contain the devices and saline water, were installed with RF transmit coil to characterize device charging capability over time. A smartphone was used to monitor the battery level. **b**, Battery voltage level after an hour of wireless charging as a function of time after immersing devices in saline water with temperature of 37 °C and 90 °C.

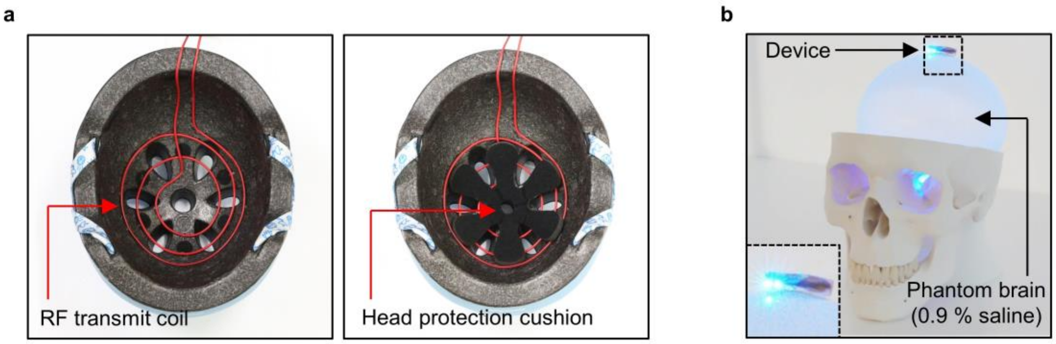

Supplementary Fig. 16 | Experimental setup to study feasibility of fully implantable, wireless rechargeable optoelectronic systems for operation in human brain. a, Optical images of a wireless charging helmet integrated with RF power transmit coil before (left) and after (right) attaching head protection cushion. **b**, Optical image of a model human head implanted with a wireless optoelectronic system. To simulate the biological environment, a phantom brain (a balloon filled with 0.9% saline water) was placed in the phantom human skull, and the device was integrated on top of the phantom brain.

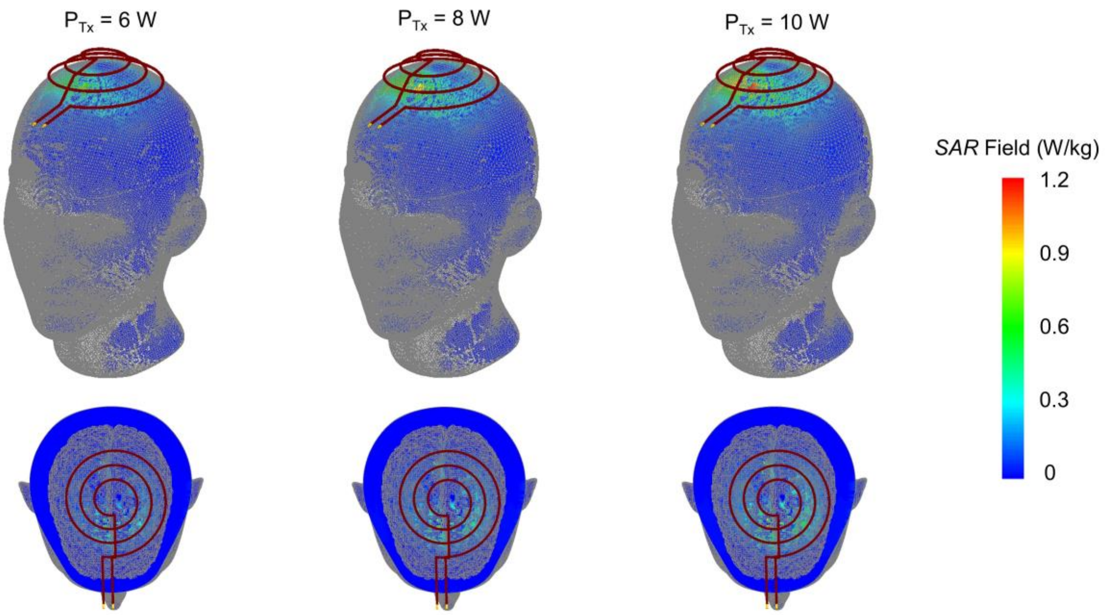

Supplementary Fig. 17 | Specific absorption rate in a human's head when wearing a wireless charging helmet supplied by different transmitting RF powers (6, 8, and 10 W).

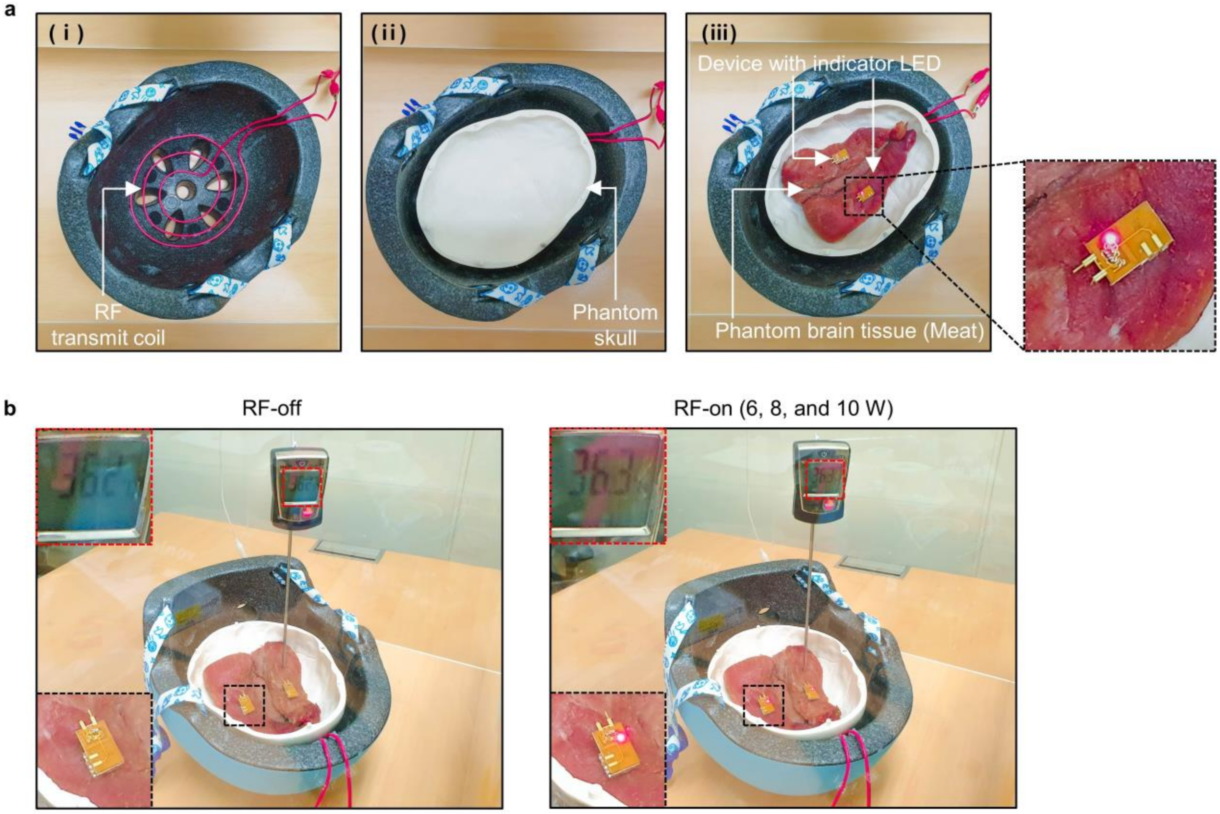

Supplementary Fig. 18 | Experimental setup to measure the temperature increase in phantom brain tissue inside a phantom skull by RF energy absorption during wireless power transfer from a wearable charging helmet. a-b, Optical images showing a phantom skull and phantom brain tissue (meat) inside a wireless charging helmet (a) and the temperature of the phantom brain tissue near the skull, measured by a probe thermometer. To mimic the biological condition, the temperature of the phantom brain tissue was maintained at 36.2 °C before applying RF power (**b**, left). The experiment simulating the wireless charging with RF input power of 6, 8, 10 W showed negligible temperature increase in the phantom brain tissue (<0.1 °C; **b**, right), proving the RF safety of our charging system.

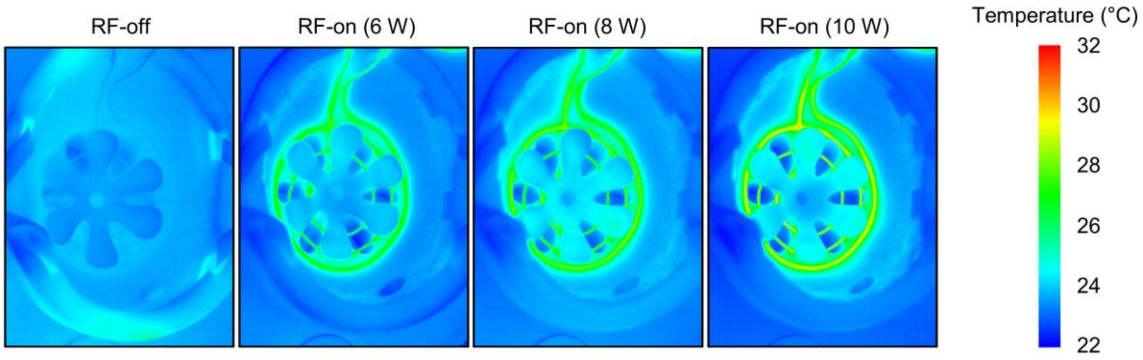

Supplementary Fig. 19 | IR images of the RF transmit coil inside a wireless charging helmet before and during wireless power transfer with different input powers (6, 8, and 10 W). The measurement was made in an ambient environment at room temperature.
